# Arousal and sustained attention fluctuate differently with respiration in younger and older adults

**DOI:** 10.1162/IMAG.a.26

**Published:** 2025-06-03

**Authors:** Ralph Andrews, Michael C. Melnychuk, Catherine N. Moran, David P. McGovern, Alexa Holfelder, Sarah Moran, Paul M. Dockree

**Affiliations:** Trinity College Dublin, Trinity Institute of Neuroscience, Dublin, Ireland; Royal College of Surgeons in Ireland, Department of Health Psychology, School of Population Health, Dublin, Ireland; School of Psychology, Dublin City University, Dublin, Ireland; Department of Old Age Psychiatry and Psychotherapy, University of Bern, Bern, Switzerland

**Keywords:** respiration, breath, attention, arousal, pupil, EEG, ageing

## Abstract

Respiration is being increasingly recognised as both synchronising its dynamics with external events and modulating internal psychophysiological states. However, the extent to which these effects stem from a respiratory modulation of attention remains underexplored. Here, we leverage differing attentional strategies of younger (YA) and older adults (OA)—OA exhibited greater focus during a simple contrast change detection task—to examine their relationship with respiratory phase-locking behaviour. OA exhibited stronger phase-locking of their respiratory cycle to task-relevant events compared with YA. Notably, participants appeared to actively adjust their breathing so that late exhalation phases coincided with target presentation, despite variable inter-target intervals. To characterise this target-locked respiratory phase window, we analysed pupil diameter and EEG frequency-power as indices of arousal and attention. Pupil diameter, frontal delta and theta, posterior alpha, and steady-state visually evoked potential (SSVEP) amplitude all varied significantly over the respiratory cycle, suggesting that arousal was enhanced for respiratory phases aligned with target expectancy and attenuated outside these phases. OA showed stronger respiratory modulation of delta, theta, and alpha, whereas YA showed stronger modulation of pupil diameter and SSVEP. We interpret these findings as evidence that respiration shapes attentional fluctuations, expanding and contracting the vigilant state across the respiratory cycle through interactions with arousal and attentional systems. Further, the age-dependent quality of attention which is applied to a task has implications for the degree of respiratory phase-locking and how physiological signatures of arousal and attention are modulated.

## 1 Introduction

Respiration oscillates throughout our life to facilitate vital gas exchange. It is also becoming increasingly apparent that the respiratory rhythm synchronises with the timing of external events and motor processes. This respiratory phase-locking seemingly ensures that certain respiratory phases in the cycle (inhalation, exhalation) are aligned with certain events. This has been reported across a range of sensory-cognitive domains: visual lexical memory (Huijbers et al., 2014), auditory oddball (Melnychuk et al., 2018), visuospatial (Perl et al., 2019), tactile perception (Grund et al., 2022), auditory perception, emotional discrimination, visual motion, and visual memory (Johannknecht & Kayser, 2022). In addition, in these studies, there was a phase-dependent modulation of task performance or sensory processing, such that it was relatively higher or lower depending on respiratory phase. Taken together, this is suggestive of the respiratory cycle acting as a synchronising mediator between environmental demands and cognitive dynamics (Heck et al., 2017,^2019^).

Presently, we suggest these diverse examples of modulation to be attentional oscillations mediated by respiration, that is, cyclic, rhythmic dilations (orientation/exploration), and contractions (focus/exploitation) of attentionally distributed cognitive resources. From this perspective, respiratory phase-locking can be viewed as an optimisation of respiratory-related cognitive and attentional biases to the dynamics of a particular sensory environment.

Here, we investigate phase-locking between respiration and task events during sustained attention in the Gradual Contrast Change Detection—Experience Sampling (GradCCD-ES) task. In this task, there is an onscreen stimulus which periodically fades in contrast and participants are instructed to respond to this target event. Additionally, the task is interrupted by thought probes which ask the participants whether they were having task-related (focused) or task-unrelated thoughts (mind wandering), assessing their mode of attention subjectively. Targets and thought probes occur after either a 3, 5, or 7 s inter-target interval (ITI), on a pseudorandom basis. These event frequencies are in a reasonable respiratory frequency range (0.14–0.33 Hz) and thus may be amenable to respiratory phase-locking. However, since there is variability in their occurrence, we are also curious to see whether respiration is dynamically adapted to accommodate this.

Respiration has also been shown to modulate pupil diameter and brain oscillations, further supporting the proposal that respiration cyclically mediates external demands with internal processing. We sought to examine the pattern of respiratory modulation of pupil diameter and brain oscillations to infer the physiological background, primarily arousal and attention related, to respiratory phases which are (and are not) synchronised with task events.

Pupil diameter is modulated by the interplay of sympathetic and parasympathetic influences. Increased sympathetic activity, linked to activation of the locus coeruleus–noradrenaline system, induces dilation whilst parasympathetic activity promotes constriction. Studies have demonstrated that moment‐to‐moment fluctuations in pupil size correlate with arousal levels, as reflected by concurrent changes in heart rate, skin conductance (Wang et al., 2018), and subjectively reported emotional arousal (Henderson et al., 2018). The strong link between pupil diameter and locus coeruleus–noradrenaline activity (Bang et al., 2023;Elman et al., 2017;Joshi et al., 2016;Meissner et al., 2024;Murphy et al., 2014) additionally implicates fluctuating attentional states. According to adaptive gain theory (Aston-Jones & Cohen, 2005), the locus coeruleus–noradrenaline system optimises task engagement by modulating neural gain, balancing exploration and exploitation. Pupil dilation, as a proxy for locus coeruleus activity, reflects shifts in attentional states, with moderate dilation linked to focused task engagement (exploitation) and larger fluctuations associated with exploratory behaviour and disengagement. Thus, variations in pupil diameter provide a physiological marker of adaptive attentional control, reflecting the dynamic regulation of cognitive resources in response to task demands. Previous studies have shown that respiratory dynamics couple to pupil dynamics (Andrews et al., 2025;Kluger, Gross, et al., 2023;Melnychuk et al., 2018,^2021^;Schaefer et al., 2023,^2024^).

Brain oscillations—frontal delta, frontal theta, and posterior alpha—further delineate fluctuating arousal and attentional states. Increased frontal delta and theta power have been associated with states of reduced external engagement, including drowsiness, mind wandering, and internally focused cognition (Kam et al., 2022). These low-frequency oscillations are thought to reflect diminished sensory processing and a relative disengagement from external task demands. Posterior alpha activity has been linked to sensory inhibition, with increased alpha power corresponding to a suppression of visual input and a shift away from external attentional demands. In the GradCCD-ES task, the stimulus flickers at 25 Hz which has been shown to evoke a steady-state visually evoked potential (SSVEP) in the visual cortex. The SSVEP, therefore, serves as a continuous marker for visual attention and sensory processing. Respiration has been shown to exhibit phase–amplitude coupling with brain oscillations in all major frequency bands, distributed across the brain (Kluger & Gross, 2021).

Observing trends in how pupil diameter and these brain oscillations alter over the respiratory cycle may provide explanatory power as to why certain respiratory phases are synchronised with certain task events.

To gain additional insight into how respiration and arousal/attention systems are coupled, we performed these analyses on two groups that have been reported to differ in their attentional strategy towards tasks: younger adults (YA) and older adults (OA).

Research utilising experience sampling thought probes during cognitive tasks has shown that OA tend to report lower levels of mind wandering than younger adults (YA) (Jackson & Balota, 2012;Jordão et al., 2019;Maillet & Schacter, 2016;Seli et al., 2017;Welhaf et al., 2024). Amongst other factors such as motivational priorities, it is thought that this age-related shift in attentional strategy may reflect a diminished cognitive flexibility, where maintaining vigilance serves as a less risky strategy than cyclically shifting between task-related and task-unrelated attentional modes.

Furthermore, we take advantage of recent findings fromMoran et al. (2025), showing that younger adults (YA) and older adults (OA) differ in their attention strategy when performing the task presently applied, the GradCCD-ES task. YA appeared to tune their attention to task targets as and when required and otherwise allow their mind to wander. By contrast, OA maintained a more focused, vigilant state throughout the task, likely a more risk-averse strategy in the face of decrements in cognitive flexibility. These conclusions were evidenced from experience sampling of self-reported focus (Moran et al., 2021,^2025^) as well as target-locked physiological indices of attention including pupil diameter, cortical event-related potentials (centro-parietal positivity, left hemisphere beta, SSVEP), and spectral power differences in EEG signal (alpha variability) (Moran et al., 2021,^2025^).

Presently, we examined respiratory-attentional coupling as a function of age. This natural division in the population provides an opportunity to study an effect of attentional strategy on respiratory phase-locking and respiratory-cyclic modulation of attentional indices.

Between the age groups, since OA seem to apply higher executive control towards the task in their more exploitative attentional strategy, we reason that their respiratory cycle should be more strongly synchronised to the task than YA. Here, we are predicting that higher vigilance will amplify synchronisation between internal respiratory rhythm and external task cues. This also aligns with the conclusion that OA entrain perceptual processes with external stimuli more strongly than YA (Lindenberger & Mayr, 2014).

The relatively continuous nature of the stimulus in the GradCCD-ES task allows mapping of the respiratory signal to physiological indices of cortical attention, continuous basic sensory processing, and pupil-linked arousal. We hypothesise that attentional indices should be greater during the respiratory phase window which is synchronised to task events. We are proposing that respiratory dynamics are organised to optimise cyclic attentional allocation. Accordingly, since we hypothesise the OA to phase-lock more strongly, we expect a more pronounced respiratory cycle-locked fluctuation in attentional indices in the OA.

Crucially, we first performed a replication analysis following the steps ofMoran et al. (2025)to confirm attentional differences between OA and YA in this paradigm. This involved an assessment of age-related differences in self-reported focus and target-locked physiological indices of pupil diameter, centro-parietal positivity (CPP; a metric of evidence accumulation prior to decision,O’Connell et al., 2012), steady-state visually evoked potential (SSVEP; 25 Hz; a continuous metric of sensory processing,Norcia et al., 2015), left hemisphere beta (LHB; 8–30 Hz; a signature of motor preparation readiness,Kilavik et al., 2013), and alpha variability (fluctuations of visual attention;Clayton et al., 2018).

Following this successful manipulation check which aligned with the conclusion fromMoran et al. (2025)that the OA exhibited a more vigilant attentional strategy, we proceeded to focus on respiration-related metrics.

## 2 Methods

The present study was approved by the Trinity College Dublin, School of Psychology Research Ethics Committee, approval ID: SPREC112020-16.

### 2.1 Participants

We aimed to replicate an age group difference in self-reported task focus. We ran a power analysis on this factor, utilising the findings fromMoran et al. (2021,^2025^). For a planned independent*t*-test, Cohen’s*d*= 0.8, alpha = 0.05, power = 0.8, power analysis recommended at least 26 participants per group. In total, 43 OA and 42 YA participated in the study (n = 85). OA were aged between 65 and 80 years, YA were between 18 and 35 years. Otherwise, inclusion criteria were no personal or family history of epilepsy, no personal or family history of unexplained fainting, no sensitivity to flickering light, no personal neurological or psychiatric illness or brain injury, normal or corrected-to-normal vision. Participants were allowed to wear glasses. A further requirement for OA was a passing score of 24 on the Montreal Cognitive Assessment (MoCA;Nasreddine et al., 2005), indicating typical cognitive functioning. OA were recruited from a research participant panel, and YA were largely students at Trinity College, recruited through college communications. Sessions lasted 2–3 h and participants were reimbursed for their time with a €40 voucher.

Eight participants were excluded due to incomplete data collection and a further five for low hit rate (>3 standard deviations from the mean). This resulted in OA n = 34, YA n = 38 for the respiratory analysis. Ten participants were excluded from this sample for the EEG analyses due to excessive noisy channels and/or high number of trials excluded during artefact rejection, OA n = 29, YA n = 33. Five OA participants were excluded from the sample for the pupil diameter analysis, OA n = 29, YA n = 38; however, additional whole blocks and trials had to be excluded due to poor quality recordings, particularly in the OA group. This resulted in an average of 307 (out of 384) trials included in the pupil diameter analysis per OA participant, and 330 trials for the YA.

### 2.2 Procedure

Participants were tested at Trinity College Dublin. Following briefing and consent, they were fitted with the EEG and respiratory belt set up, OA filled in the Montreal Cognitive Assessment, both groups were given instructions for the GradCCD-ES task (see next section), and they performed the said task for eight blocks, approximately 8 min each. Additionally, all participants filled out pre- and post-task subjective state questionnaires which are not reported here.

### 2.3 Gradual contrast change detection with experience sampling (GradCCD-ES) task

Participants performed the GradCCD-ES task on a 40 cm cathode-ray tube presentation monitor in a sound-attenuated room with no lighting except the monitor. Participants sat at a distance of approximately 57 cm from the monitor, with their head supported by a chinrest to minimise head and eye movements. The monitor was set to a 100 Hz refresh rate and resolution of 1024 x 768. The task was hosted on MATLAB 2016b using the Psychtoolbox-3 toolbox (Brainard, 1997). Prior to the task, participants were given verbal and visual task instructions as well as information on mind wandering as they were required to indicate during the task whether they were “focused on the task,” or “mind wandering” (experiencing task-unrelated thoughts). They additionally performed a short practice round with the experimenter in the room, to demonstrate features of the task and assess comprehension.

**Fig. 1.**
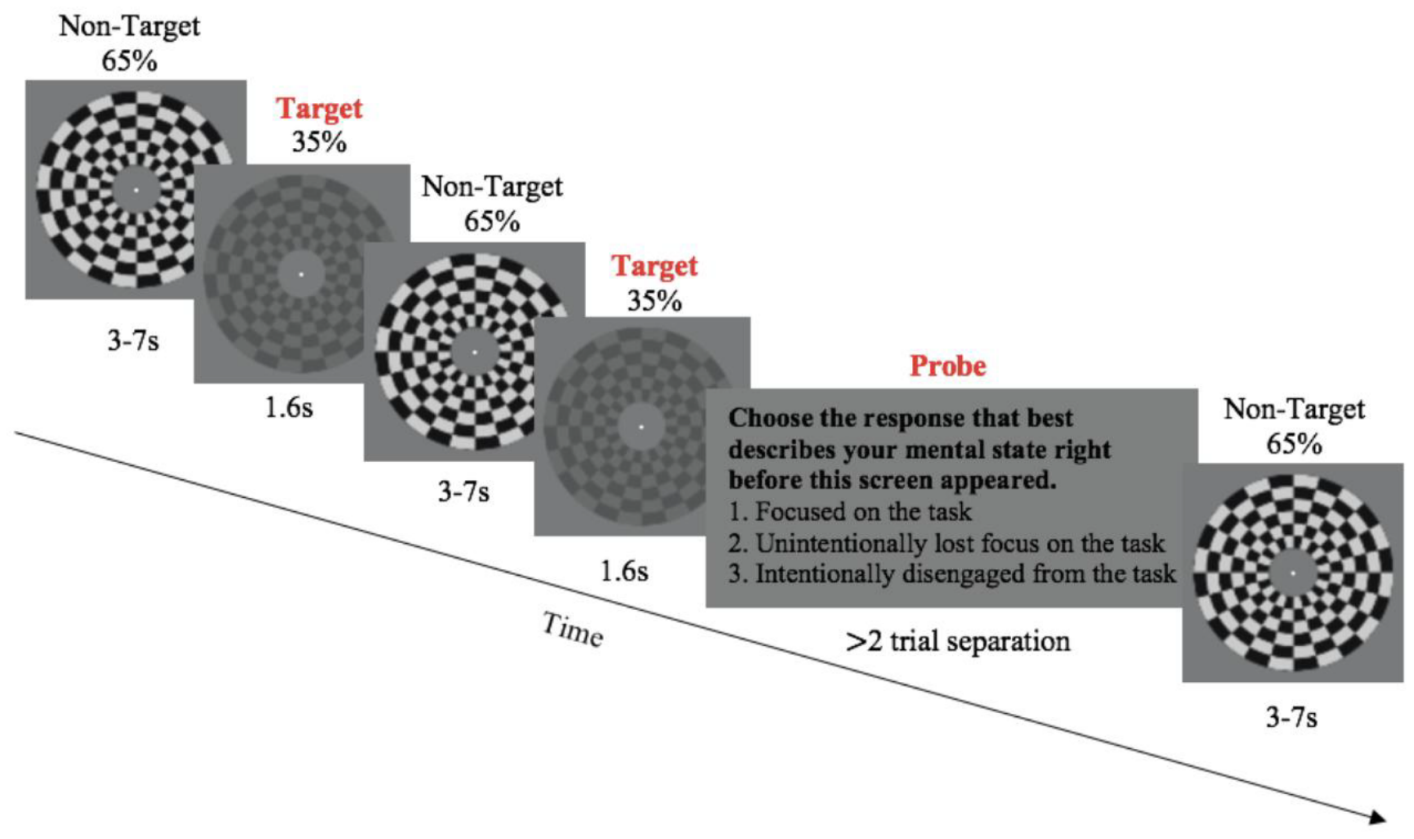
Task schematic of the Gradual Contrast Change Detection with Experience Sampling (GradCCD-ES) Task. A continuously presented checkerboard, anulus stimulus decreases in contrast after variable intervals which is the target to be identified by participants, who respond with a mouse click upon recognition. Additionally, the stimulus is interrupted with experience-sampling thought probes (TPs) which ask the participant to categorise their attentional state immediately prior to the TP presentation. Each block consists of 48 targets, 16 TPs, and lasts approximately 8 min each. A testing session consisted of eight blocks.

During the GradCCD-ES task (McGovern et al., 2018;Moran, 2021;Moran et al., 2025;O’Connell et al., 2012), participants fixated on the centre of the centrally presented, black and white, checkerboard annulus stimulus (outer radius = 8°, inner radius = 3°) displayed on a grey background (Fig. 1). The stimulus flickered on and off at a frequency of 25 Hz, to induce a steady-state visually evoked potential (SSVEP) observable in the EEG over occipital areas at the same frequency. This served as a continuous signal indicating sensory encoding of the stimulus. The target to be identified in the task was a gradual decrease in stimulus contrast (65% to 35% over 1.6 s), which then returned to its original contrast (over 0.8 s). Participants were instructed to respond with a mouse click (right hand) as soon as they noticed this target. Targets occurred after variable inter-target intervals of 3, 5, and 7 s, with the order of intervals shuffled randomly within each block. There were 48 targets in each block. Additionally, the continuous stimulus presentation was interrupted periodically by the presentation of “thought probes” (TPs), with the same variable pseudorandom intervals (3, 5, or 7 s). However, they occurred 16 times per block (>2 target trial separation). Here, the stimulus was taken off-screen for 500 ms and replaced with text stating: “Choose the response that best describes your mental state right before this screen appeared. (1) Focused on the task, (2) unintentionally lost focus on the task, or (3) intentionally disengaged from the task.” The intentionality of mind wandering (responses “2” and “3”) was of interest toMoran et al. (2025), who followedSeli et al. (2015), and we preserved the paradigm for replication purposes, however, the intentionality was not of interest to the current investigation. Thus, these responses are combined into “mind wandering.” Participants responded with their perceived attentional state immediately prior to the TP presentation with a keyboard press of “1,” “2,” or “3” (right hand). Each block consisted of 48 targets, 16 TPs, and lasted ~8 min each. Participants performed eight blocks in the session and were offered short breaks in between every block.

From participant responses we derived: (i) mean reaction time (RTm; time period between target presentation and participant response), (ii) coefficient of variation of RT (standard deviation of RT divided by RTm), (iii) hit rate (proportion of identified targets), (iv) false alarms; (clicks outside of target presentation time, and not counted as a late response which were clicks made 2200–2400 ms after target presentation), and (v) focus/mind wandering (proportion of TP responses indicated). Each behavioural variable was averaged after collapsing trials across the blocks, and focus/mind wandering counts were averaged across the blocks.

### 2.4 Pupillometry acquisition and preprocessing

Pupil diameter was collected using Eyelink 1000 SR Research camera and associated software from the left eye at 1000 Hz. Participants were instructed to maintain their head on a chinrest to minimise pupil detection loss. A calibration and validation check was performed before each block.

Data were pre-processed using custom-made MATLAB scripts. For blink detection, the blink indices provided by the Eyelink automatic detection were extended 50 ms either side. These periods were removed and interpolated linearly. Blink/interpolated sample points within a recording session were saved and used for trial rejection in subsequent analyses, excluding trial windows that contained >30% blink/interpolated samples. Pupil diameter was then low pass filtered using a zero-phase digital Butterworth filter with cut-off frequency 4 Hz and filter order 4, and linearly detrended.

Pupil diameter was used in multiple analyses: pre- (-2000 ms) and post-target (+4000 ms) amplitude to infer age-related differences in line withMoran et al. (2021,^2025^), peri-target (-7000 to +7000 ms) pupil phase to infer target anticipation behaviour, and the amplitude of pupil diameter over the average respiratory cycle.

Target-locked windows were extracted and checked for number of blink/interpolated samples. Pre-target windows were end corrected by subtracting the mean value 200 ms post-target, and post-target windows were baseline corrected subtracting 200 ms pre-target. Subtractive baselining is generally preferred over divisive to minimise data distortion (Mathôt et al., 2018). For the peri-target window, we isolated phase information from amplitude. We performed “empirical mode decomposition” (EMD) on pupil data to obtain “intrinsic mode functions” (IMFs) which are oscillatory components extracted from non-stationary signals (like pupil diameter). We chose the first IMF (contains the highest frequency component) as it provided a solid phase representation when the Hilbert transform was applied to obtain instantaneous phase. Instantaneous phases of pupil diameter were extracted for the peri-target period and then cosine transformed to provide a waveform shape for plotting a visual representation. Pupil diameter amplitude over the respiratory cycle was calculated using a moving phase window and is described alongside other variables in section “Physiological indices over the respiratory cycle.”

### 2.5 EEG acquisition and preprocessing

#### 2.5.1 Acquisition and preprocessing

EEG signal was collected using Biosemi equipment and software with 128 scalp electrodes plus 2 electrooculogram electrodes (above and below the left eye) recorded at 256 Hz. Data were pre-processed using custom-made MATLAB scripts and FieldTrip toolbox (Oostenveld et al., 2010). Data were detrended, band-pass filtered at 0.2–40 Hz, with two pass method and hamming window, and re-referenced using global average. The analysis involving beta frequency was also band-stop filtered at 23–27 Hz to exclude the 25 Hz stimulus flicker which generated the steady-state visually evoked potential. Channels deemed to have excessive variance by visual inspection were removed and interpolated using spherical spline method. Participants with number of interpolated channels >10% were excluded.

#### 2.5.2 Target-locked preprocessing

Target-locked neural indices of cognitive processing were generated in order to replicate age-related differences in attentional strategy and follow the steps outlined byMoran et al. (2021,^2025^). EEG data were extracted -250 to 2000 ms with respect to target presentation for the post-target centro-parietal positivity (CPP), steady-state visually evoked potential (SSVEP, 25 Hz), and left hemisphere beta (LHB, 8–30 Hz). Trial windows were -2000 to 0 ms relative to target for pre-target alpha. Trials were baseline corrected by subtracting the mean within the first 250 ms period. Each trial was subject to artifact detection using thresholds of 250 μV for eye electrodes (upper minus lower), and 100 μV for scalp electrodes, rejecting all trials where any values exceeded these values. Miss trials were also excluded. Finally, current source density was performed to increase spatial resolution (Kayser & Tenke, 2015).

#### 2.5.3 Frequency–power preprocessing

For the subsequently described “*Physiological indices over the respiratory cycle”*analysis, EEG power in delta (1–4 Hz), theta (4–7 Hz), alpha (8–14 Hz), and SSVEP (25 Hz) frequency bands were analysed. EEG data for this were subject to the same above preprocessing steps prior to trial segmentation. Frequency–power across each block was calculated using short-time Fourier transform, window size 1 s with 20% overlap, which was then z-score normalised. The corresponding median respiratory phase angle within each window was additionally extracted. Channels chosen for each frequency band were determined from grand-average population mean topographic plots as those showing the highest power. Delta and theta channels: FP1, FP2, FPz; alpha channels: P07, P7, P8; SSVEP channels: PO4, PO7 (see Supplementary Materials,Fig. S5).

### 2.6 EEG target-locked neural indices analysis

Target-locked neural indices of cognitive processing were generated in order to replicate age-related differences in attentional strategy and follow the steps outlined byMoran et al. (2021,^2025^).

#### 2.6.1 CPP

Group average topographic plots -150 to -50 ms relative to target response showed a widespread area of positivity which varied between participants and thus CPz was chosen to be consistent withMoran et al. (2021,^2025^). Trials were averaged for each participant at channel CPz and then across participants in each group to generate the CPPs. CPP slope was calculated for a linear best fit line 250 to 750 ms of the participants mean post-target. CPP peak was the maximum value of the participant mean 500 to 1750 ms post-target. Latency was the time point post-target of the CPP peak. For plotting, CPPs were low-pass filtered using a zero-phase digital Butterworth filter with cut-off frequency 8 Hz and filter order 4. The CPP onset latency plotted was calculated using a running*t*-test and determined as the first sample point to significantly deviate from 0 in a positive direction (seeLoughnane et al., 2016,^2019^).

#### 2.6.2 SSVEP

Channel selection for the SSVEP was determined by signal-to-noise ratio group average topographic plots of the power at 25 Hz divided by the mean power of neighbouring frequencies of the fast Fourier transform, 23 and 27 Hz. Highest power was observed for OA at POz, and YA at PO4. For each trial, a short-time Fourier transform of 400 ms window size (8 cycles of SSVEP) and 20 ms step size was applied, which were then averaged for each participant. Participant means were baseline normalised by dividing by the mean value in the first 250 ms. SSVEP slope was calculated for a linear best fit line 350 to 850 ms of the participant mean post-target. SSVEP amplitude was calculated as the mean value 500 to 1600 ms of the participant mean post-target.

#### 2.6.3 LHB

Channel selection for the LHB was determined from group average topographic plots as the electrode with the minimum power -100 to 100 ms in the 8–30 Hz range (25 Hz SSVEP power band-stop filtered out) relative to target response—C3 for both groups. For each trial, a short-time Fourier transform of 400 ms window size and 20 ms step size was applied, which were then averaged for each participant. Participant means were baseline normalised by dividing by the mean value in the first 250 ms. LHB slope was calculated for a linear best fit line 350 to 850 ms of the participant mean post-target. An additional LHB Slope variable was determined around the time of mean RT, in the window 1000 to 1300 ms post-target. LHB amplitude was calculated as the mean value 500 to 1250 ms of the participant mean post-target.

#### 2.6.4 Alpha variability

Topographic plots revealed channels PO6 and PO8 and channels PO5 and PO7 to show the maximal amplitude of alpha -2000 to 0 ms relative to target onset for the younger and older adults, respectively. Short-time Fourier transform was performed over the pre-target period with a 400 ms window and 20 ms step within the alpha (8–14 Hz) frequency band. Between-trial variability was calculated as the coefficient of variation of alpha amplitude; the standard deviation divided by mean alpha amplitude.

For the neural indices’ statistics and plots, we ensured that a sufficient number of trials were included for each participant, to contribute to a high signal-to-noise ratio (Supplementary Materials,Table S4).

### 2.7 Respiration acquisition and preprocessing

#### 2.7.1 Recording and preprocessing

Respiratory signal was collected using a SleepSense respiratory effort sensor belt, recorded at 256 Hz via a Biosemi Amplifier and Biosemi Actiview software. Data were pre-processed using custom-made MATLAB scripts. Signals were first inspected by eye for poor recording sessions and then were low pass filtered using a zero-phase digital Butterworth filter with cutoff frequency 0.6 Hz and filter order 4. Finally, filtered signals were then linearly detrended.

### 2.8 Respiration-task event phase-locking

We performed “empirical mode decomposition” (EMD) on processed respiration data to obtain “intrinsic mode functions” (IMFs) which are oscillatory components extracted from non-stationary signals (like respiration). We chose the first IMF (containing the highest frequency component) as it provided a clear phase representation when the Hilbert transform was applied to obtain instantaneous phase. The instantaneous phase angles in radians were extracted at the times of task events—target presentation, target response, TP presentation, TP response. These event-locked phase angles were collapsed across all blocks and tested against the null hypothesis of uniformity with a Rayleigh’s test. The resultant vector length of respiratory phase angles was the primary measure of phase-locking as well as the mean angle it tended towards. Circular statistics were performed using CircStat toolbox (Berens, 2009). SeeCremers and Klugkist (2018)for more information on calculating circular statistics.

### 2.9 Physiological indices over the respiratory cycle

To test for a modulation of other physiological signals over the respiratory cycle, we considered pupil diameter and EEG power in delta (1–4 Hz), theta (4–7 Hz), alpha (8–14 Hz), and SSVEP (25 Hz) frequency bands.

The moving phase bin window analysis involved choosing a phase window length and the phase window overlap, and computing the mean of the variable within each phase bin, moving around the whole respiratory cycle. For EEG powers (see*EEG Acquisition and Preprocessing*for prior preprocessing): phase window length = π/36, window overlap = 75%; pupil diameter (see section*Pupillometry Acquisition and Preprocessing*for prior preprocessing): phase window length = π/36, window overlap = 75%. Plots were corrected by shifting the resultant mean so that the plotted point aligns with the median phase of the phase bin (rather than the start). The phase window size, divided by the phase step size, divided by 2, gave the number of sample points the data should be shifted by.

To determine the significance of variable means within any given respiratory phase bin, one-sample permutation testing was used: for each phase bin, the corresponding actual values were permuted 10,000 times, such that in each permuted distribution, they were randomly attributed positive and negative signs and the mean was taken. The corresponding*p*value for each phase bin was calculated as the percentage of permuted means that were higher or lower than ± actual phase bin mean. The corresponding*t*statistic was the actual phase bin mean minus the mean of permuted means, over the standard error of the mean (SEM) of the actual phase bin. Statistical significance was determined at the*p*= 0.05 level for all variables. Resulting significant phase bins were subsequently reassessed with a false discovery rate (FDR) procedure to control for false positives due to the large number of tests across the respiratory cycle. The*p*-values were ranked from smallest to largest, and for each rank, the*p*-value was compared with an FDR threshold, calculated by multiplying the*p*-value rank by the desired FDR level (0.05) and dividing by the total number of tests. Original*p*-values that were smaller than or equal to their corresponding calculated threshold were considered still significant (Benjamini & Hochberg, 1995).

## 3 Results

### 3.1 Age group differences in behavioural, experiential, and physiological signatures of attention

We first performed the same analysis asMoran et al. (2025)to replicate and establish that our age groups of younger adults (YA) and older adults (OA) differed in their attentional strategy towards the task. Our findings comparing task-derived and physiological indices of attention indicate that the OA had higher attentiveness towards the task than the YA, drawing the same conclusion asMoran et al. (2025). A full analyses of task differences with figures to illustrate are provided in the Supplementary Materials.

OA exhibited significantly higher self-reported focus in response to thought probes (*t*(70) = 4.83,*p*< 0.001,*d*= 1.14) and lower pre-target alpha variation (*U*= 327,*p*= 0.03,*r*= -0.32), indicating more stable engagement with the task than YA. During the target trial itself, OA exhibited a lower slope in the steady-state visually evoked potential (SSVEP) than YA (*t*(60) = -2.48,*p*= 0.016,*d*= 0.27), indicating more adept tracking of the sensory evidence for contrast change. Additionally, near the response time OA exhibited a lower left hemisphere beta (LHB) slope (*t*(60) = -2.86,*p*= 0.006,*d*= -0.73), indicating a more pronounced motor preparation. Finally, OA showed higher post-target pupil diameter than YA (*t*(65) = 3.63,*p*< 0.001,*d*= 0.90), indicating greater modulation of the arousal system to goal-relevant information.

In contrast, there were no significant differences in task performance between OA and YA when comparing reaction time,*t*(70) = 1.23,*p*= 0.22, reaction time coefficient of variation,*t*(70) = -1.10,*p*= 0.38, hit rate,*t*(70) = -0.47,*p*= 0.64, false alarm,*t*(70) = 0.73,*p*= 0.47.

For the crucial age group difference in self-report focus, we ran a post hoc Bayesian robustness check to assess how the support for the result evolved as a function of increasing sample size. Evidence for a significant difference was supported after n = 40 through to the full n = 72. Final Bayes factor = 2110.

This successful replication analysis permitted proceeding with respiratory-related analyses under the position that the OA showed a more persisting attentive stance towards the task than the YA, despite comparable task performance.

### 3.2 Respiratory phase-locking to task events

Working under the evidenced assumption of an age group difference in attentional strategy during the Gradual Contrast Change Detection—Experience Sampling (GradCCD-ES) task, we sought to investigate how these groups may also differ in how respiration phase-locks to task events.

Having transformed the respiratory waveform into its instantaneous phase, we considered the distribution of respiratory phases which were occurring at the time of task events—target presentation, target response, thought probe (TP) presentation, TP response, for each participant.

**Fig. 2.**
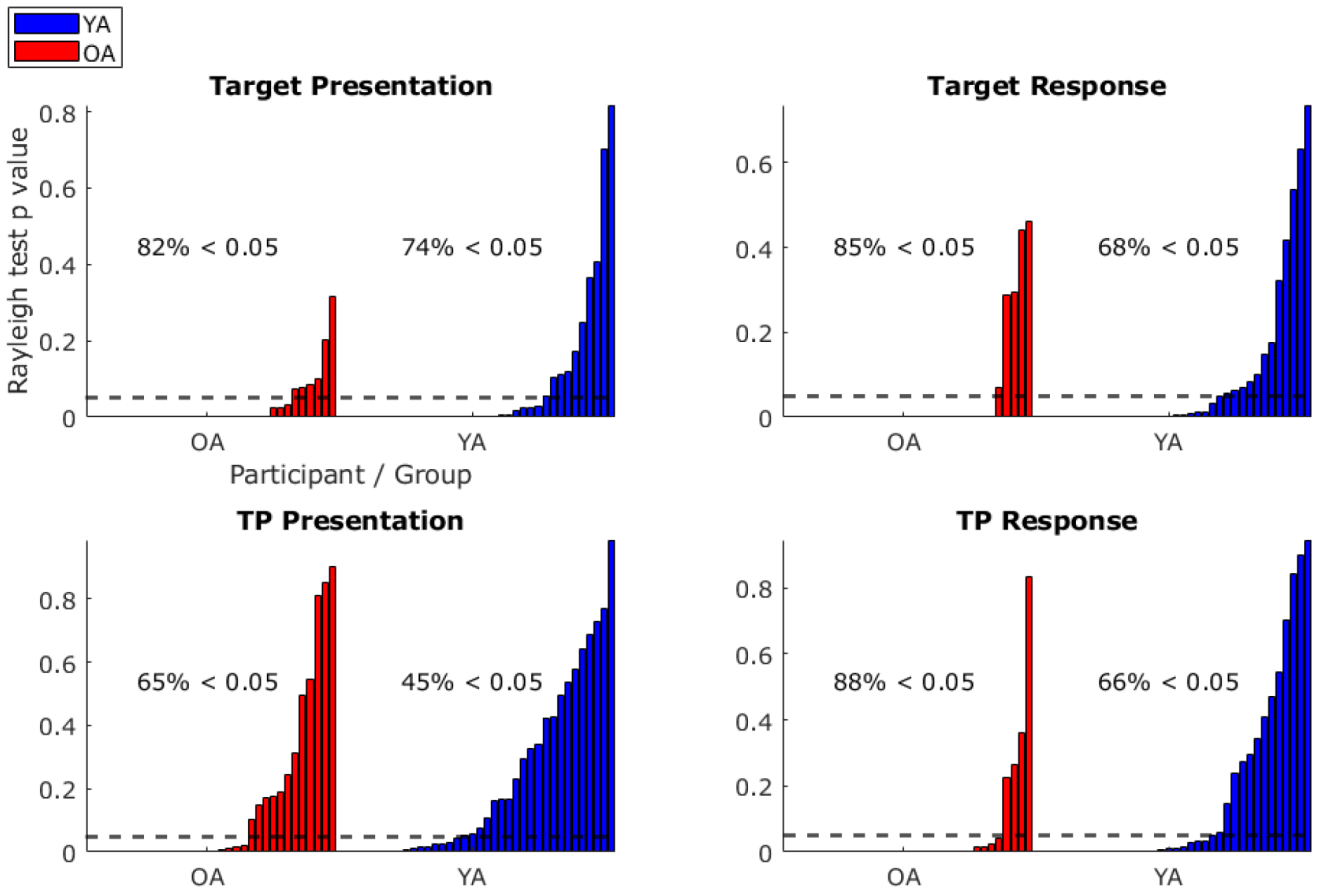
Bar graphs of*p*values returned from Rayleigh tests against a null hypothesis of uniformity for respiratory phase angles at the times of target presentation, target response, thought probe (TP) presentation, and TP response. Older adults (OA) in red, younger adults (YA) in blue. Values displayed on graphs indicate the percentage of participants in each group that showed significant (*p *< 0.05; dashed line) non-uniformity. Significant non-uniformity was a majority group finding (except YA TP presentation), but not an ubiquitous one.

The percentage of participants’ respiratory phase distributions which rejected the Rayleigh test null hypothesis of uniformity (and, therefore, a respiratory phase preference) were as follows: target presentation: OA*M*= 82%, YA*M*= 74%; target response: OA*M*= 85%, YA*M*= 68%; TP presentation: OA*M*= 65%, YA*M*= 45%; TP response: OA*M*= 88%, YA*M*= 66% (Fig. 2). Respiration-task event phase-locking was, therefore, a majority group finding (except for the YA TP presentation), but not a ubiquitous one.

The primary measure of respiration-task event phase-locking was the resultant vector length (VL) of respiratory phase angle distributions. A higher VL indicates lower variability in phase angles (and hence stronger phase preference). OA had significantly higher VL for target presentation, OA*M*= 0.18 ± 0.01, YA*M*= 0.14 ± 0.01,*t*(70) = 2.10,*p*= 0.04,*d*= 0.50; target response, OA*M*= 0.23 ± 0.02, YA*M*= 0.15 ± 0.01,*t*(70) = 3.69,*p*< 0.01,*d*= 0.87; TP presentation, OA*M*= 0.22 ± 0.02, YA*M*= 0.15 ± 0.01,*t*(54.79) = 2.76,*p*< 0.01,*d*= 0.66; and TP response, OA*M*= 0.45 ± 0.05, YA*M*= 0.24 ± 0.03,*U*= 977,*p*< 0.01,*r*= 0.51 (Table 1). When correcting for multiple comparison for these four tests, an adjusted alpha of 0.0125 (0.05/4) leaves the target presentation result insignificant. Overall, the OA showed more consistent respiratory-task event phase-locking.

**Table 1.** Results from means comparisons tests between older adults (OA) and younger adults (YA), testing for differences in respiratory-task event vector lengths (VL; measure of respiratory phase-locking variability).

**Respiratory-Task Event Vector Lengths (VL) Age Group Comparisons**				
**Independent Samples T-Test**				
	***t***	**df**	***p***	**Cohen’s** ***d***
Target Presentation VL	2.10	70	0.04	0.50
Target Response VL	3.69	70	**<0.01**	0.87
TP Presentation VL ^a^	2.76	54.8	**<.001**	0.66

**Mann–Whitney U-Test**				
	***U***		***P***	**Rank-Biserial Correlation**
TP Response VL	977		**< 0.01**	0.51

OA had significantly higher VL for target response, thought probe (TP) presentation, and TP response, when correcting significance threshold for multiple comparisons (*p*< 0.0125). Thus, OA, showed a greater consistency in which respiratory phases aligned with these task events.^a^Welch’s T-Test.

For the age group difference in VL, we ran post-hoc Bayesian robustness checks to assess how the support for the results evolved as a function of increasing sample size (up to the full sample size of 72). Evidence for a significant difference was supported for target presentation only after n = 70, final Bayes factor (BF) = 1.56. Target response after n = 44, final BF = 61.21, TP presentation after n = 48, final BF = 6.80, TP response after n = 44, final BF = 284. Therefore, these results showed considerable robustness except for target presentation.

**Fig. 3.**
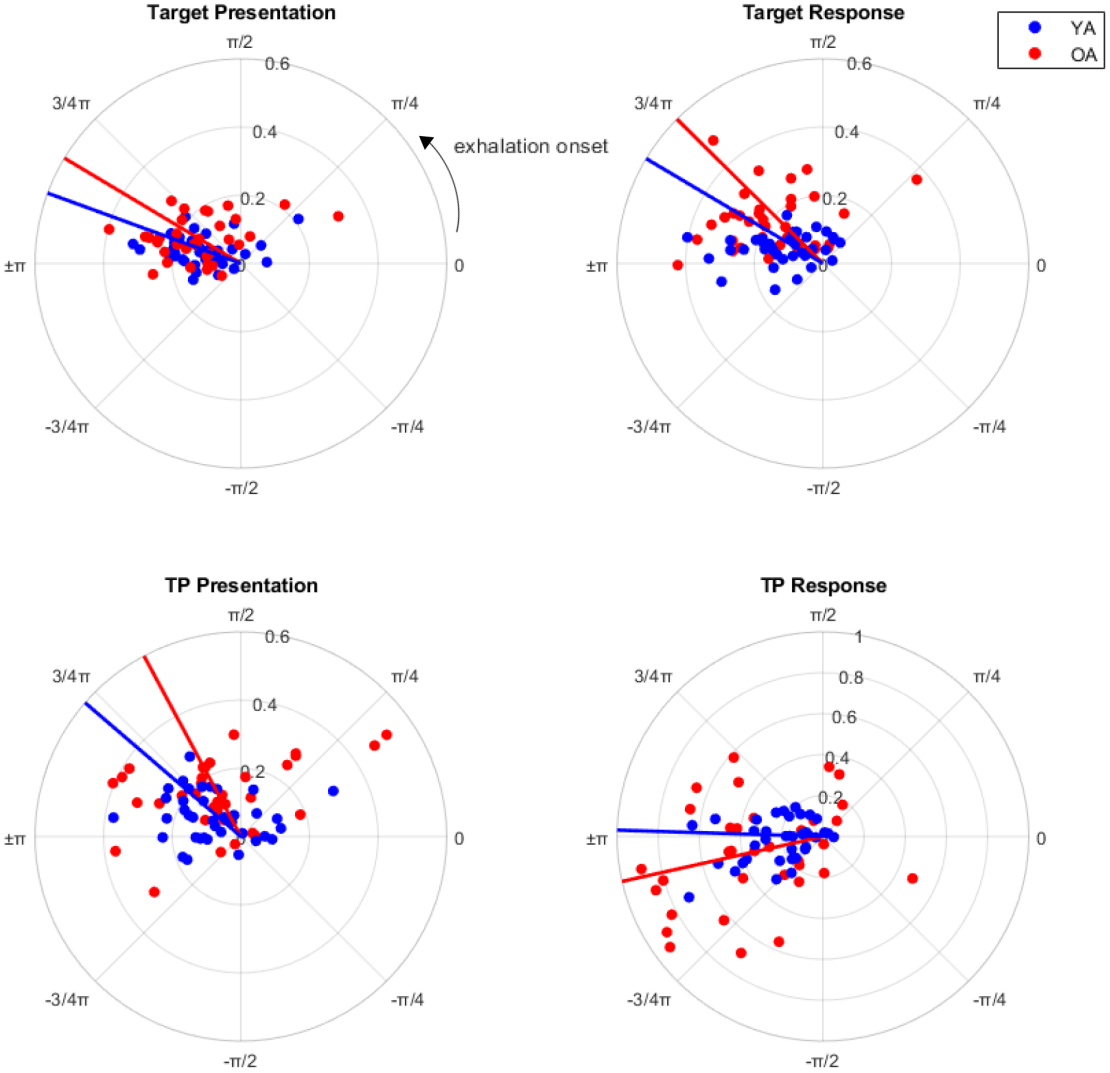
Polar scatter graphs of the mean resultant vector lengths (VL; radial axis) and respiratory phase angles (angular axis in radians), indicating the strength and angle of respiratory-task event phase-locking, respectively. Dots represent participant means and solid lines represent the direction of the group mean angles, older adults (OA) in red, younger adults (YA) in blue. 0 radians represents exhalation onset, and the respiratory cycle proceeds anti-clockwise. The majority of respiratory phase angles occurred between mid-exhalation and early inhalation.

Figure 3contains circular plots showing the mean respiratory phase angle distributions for all participants at the time of task events and the respective VL. The mean respiratory angles for target presentation (OA*M*= 2.60 rad, YA*M*= 2.79 rad), target response (OA*M*= 2.36 rad, YA*M*= 2.60 rad), and TP presentation (OA*M*= 2.06 rad, YA*M*= 2.43 rad) fell within the latter half of exhalation (π/2 to π). TP response (OA*M*= -2.92, YA*M*= 3.11) was more aligned with the exhalation-to-inhalation transition (±π). The mean direction of respiratory phase distributions did not significantly differ between age groups—all*p*values > 0.12 from Watson–Williams tests.

### 3.3 Evidence of adaptive respiratory behaviour to task events

From our respiratory circular phase plots (Fig. 3), we observed that target presentation, target response, and TP presentation were majorly aligned with participants’ late exhalation phases. TP response was aligned with early inhalation. We decided to test for evidence that participants were actively adapting their respiratory dynamics to facilitate this alignment, particularly the OA group who showed stronger phase-locking.

The GradCCD-ES task presents targets at either 3, 5, or 7 s inter-target intervals (ITI), on a pseudorandom basis. Therefore, for participants to align a specific respiratory phase, this would require adaptive changes to respiratory behaviour to meet these changing ITI.

Firstly, a mixed-model ANOVA indicated that there was no significant difference in respiration-target vector length (VL; angular variability) across ITI,*F*(1.99) = 2.14,*p*= 0.12, indicating that respiratory phase-locking occurred with a similar strength for each. A Harrison–Kanji test (circular mixed-model ANOVA) for difference in the mean phase angle of respiratory-target phase-locking showed a significant main effect of ITI, X^2^(2) = 11.42,*p*< 0.01, no main effect of group, X^2^(4) = 7.97,*p*= 0.09, and no interaction, X^2^(2) = 4.23,*p*= 0.94 (circular plots shown in Supplementary Materials,Fig. S3). Significant differences in angle direction lay between 3 and 7 s,*F*(1) = 8.39,*p*< 0.01, and 5 and 7 s,*F*(1) = 4.20,*p*= 0.04. The mean angle for target alignment for the 7 s ITI was 2.97 rad, compared with 2.46 rad for 3 s and 2.57 rad for 5 s. Thus, although the 7 s angles were significantly later in the respiratory cycle (closer to exhalation-to-inhalation transition), they were not dramatically so.

To give a depiction of grand-average respiratory phase changes over the target period, we calculated the instantaneous phases of respiration 7000 ms either side of target presentation and then calculated the cosine of this for an amplitude-agnostic phase waveform for each participant. As shown inFigure 4a, there is a distinct pre-target and post-target inspiratory peak for each ITI. The post-target peak is consistently at +3900 ms and seemingly represents a robust response, preceded by a consistent exhalation at +2200 ms. The pre-target peak is, therefore, likely to be the response from the previous target, which occurred at -1363, -3398, and -5355 ms for the 3, 5, and 7 s ITI, respectively (age combined). These inspiratory peaks, therefore, occurred with approximately 2000 ms difference with each 2000 ms increase in ITI. In between these robust responses, the phase shifts appear to be more inconsistent between participants, represented by the flatter curve and wider standard error of the mean (shaded area). These phase patterns are more pronounced in the OA, indicating more consistent within-group phase changes.

**Fig. 4.**
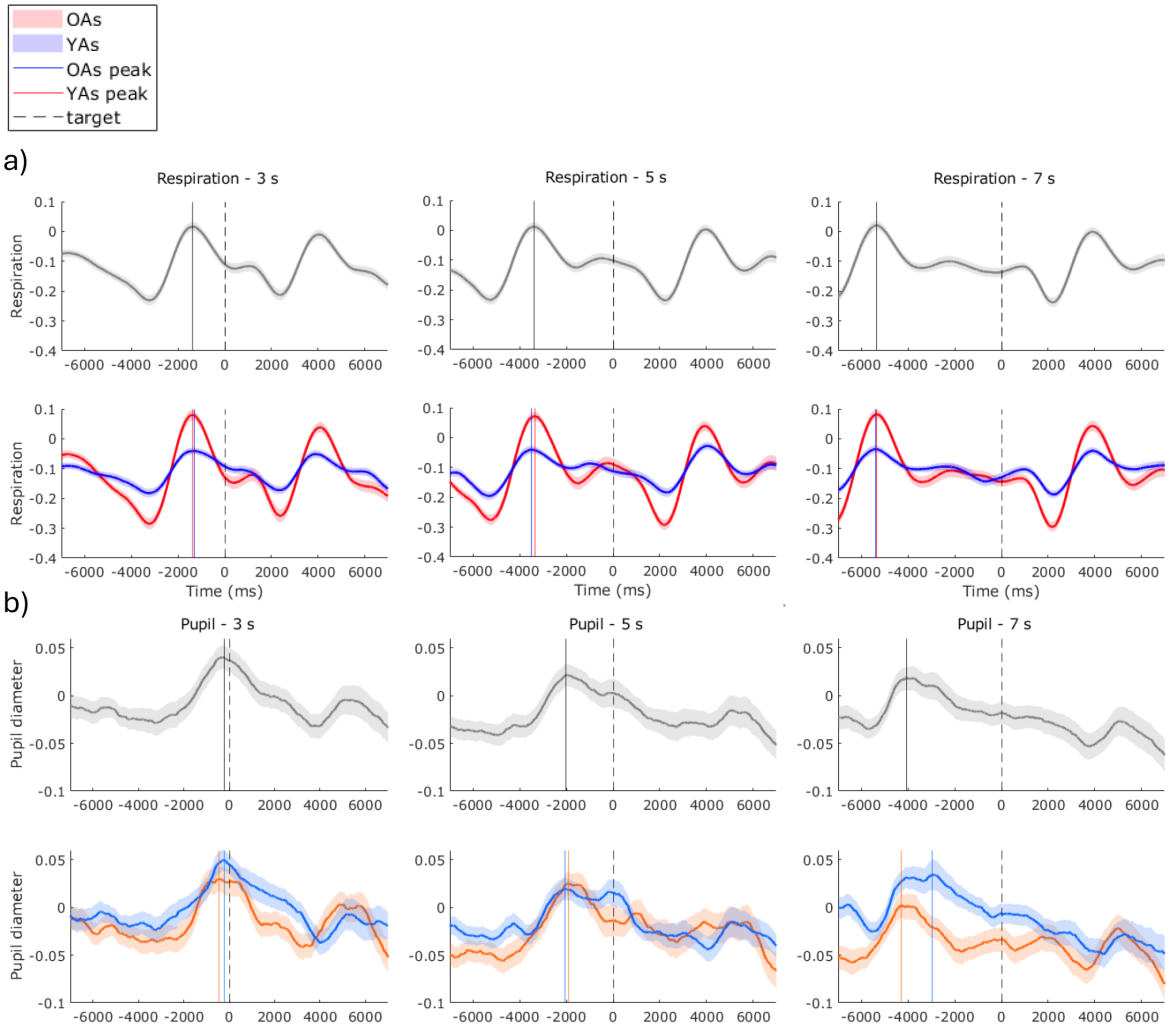
Grand-averaged (a) respiration and (b) pupil diameter, -7000 ms pre- and -7000 ms post-target presentation, for each of the inter-target intervals (3, 5, 7 s). Mean respiration and pupil diameter were calculated as the instantaneous phases over the target period, then cosine transformed to give an amplitude-agnostic, phase waveform. Top plots (black) show grand averages of all participants and bottom plots show grand averages split for older adult (OA; reds) and younger adult (YA; blues) groups. Lighter colours either side of mean represent the standard error of the mean (SEM). Vertical dashed line represents target presentation time. Vertical solid lines mark the time of the pre-target peak.

Adapting the respiratory cycle to phase-lock with changing ITI should considerably alter respiratory dynamics. Thus we assessed Pearson’s correlations between the target presentation vector length (consistency of phase-locking) and respiratory rate mean (RRm), RR coefficient of variation (RRcv), RR autocorrelated variability (RRar). RRcv represents the*total*variability in RR, whereas RRar represents the*structured*variability in RR. From these metrics, one can infer variability and its structuredness. This analysis approach was developed by Vlemincx and colleagues (e.g., seeVlemincx et al., 2012). With three correlations, alpha was adjusted to 0.05/3 = 0.017 and the effect of age group was controlled for. There was a significant positive correlation between target vector length and RRcv,*r*(70) = 0.44,*p*< 0.001, and negative correlations with RRm,*r*(70) = -0.61,*p*< 0.001, and RRar,*r*(70) = -0.51,*p*< 0.001. In other words, as respiratory-target phase-locking consistency increases, RR is slower, and total variability in RR increases, but the structured variability decreases, and thus it can be inferred that random variability increases. These correlations imply that maintaining respiratory-target phase-locking to changing ITI requires inducing considerable random variability into respiratory rate.

Mean respiratory frequency did not significantly differ between the age groups, OA*M*= 0.21 Hz ± 0.02, YA*M*= 0.25 Hz ± 0.01,*t*(70) = -1.65,*p*= 0.1. Thus, it is unlikely that age-related differences in habitual respiratory frequency produced changes in respiratory phase-locking.

All in all, we take these analyses as supporting evidence for participants’ adapting their respiratory dynamics to align late exhalation—early inhalation phases with target presentation despite changing ITI.

As a further exploratory analysis, we asked whether respiratory-target entrainment was a time-dependent phenomenon by assessing changes in target VL over the task. A mixed-model ANOVA of target VL across the eight blocks showed no significant main effect of blocks,*F*(7) = 0.38,*p*= 0.92, a significant main effect of group,*F*(1) = 5.06,*p*= 0.03, and no interaction,*F*(7) = 1.71,*p*= 0.10. A mixed-model ANOVA comparing the four quartiles after averaging across the blocks indicated no significant main effect of quartiles,*F*(2.90) = 0.55,*p*= 0.65, no main effect of group,*F*(1) = 3.29,*p*= 0.07, no interaction between them,*F*(3) = 2.57,*p*= 0.06. The trend towards an interaction appears to be driven by a decrease in VL for OA and increase in VL for YA in the last quartile (Supplementary Materials,Fig. S4).

### 3.4 Pupil diameter over the target presentation

The results in the previous section suggest the possibility of an anticipatory respiratory adaption so that target presentation is aligned with late exhalation phases. Pupil diameter serves as a useful indicator of arousal and attentional state. We looked at pupil diameter phase over the target presentation period to see whether pupil diameter indicated any kind of target anticipation from the arousal system akin to that of the respiratory system.

Pupil diameter phase -7000 to 7000 ms over the target presentation is plotted inFigure 4b. After a 3-s ITI, pupil diameter is at its maximum almost precisely at the time of target presentation, -230 ms. After a 5-s ITI, pupil diameter peaks at -2012 ms relative to target, and after a 7-s ITI, pupil diameter peaks at -4063 ms. Pupil diameter gradually declines following the pre-target peak and then has a consistent response across the ITI, it sharply rises again around +4000 ms. Similar to the pre-target respiratory peaks, the pupil diameter peaks were at approximately 2000 ms intervals according to the ITI and then there was a consistent robust response/anticipation of the next target.

### 3.5 Physiological indices over the respiratory cycle

In this analysis, we sought to characterise pupil diameter and electroencephalography (EEG)-derived frontal delta, theta, and posterior alpha oscillations, over the average respiratory cycle. These physiological signals are commonly implicated in fluctuating arousal and attention states. By observing how they fluctuate across the respiratory cycle, we sought to better understand the background physiological states that accompany the respiratory behaviours outlined above. We also analysed the steady-state visually evoked potential (SSVEP), generated in the visual cortex by the onscreen stimulus flickering continuously at 25 Hz.

**Fig. 5.**
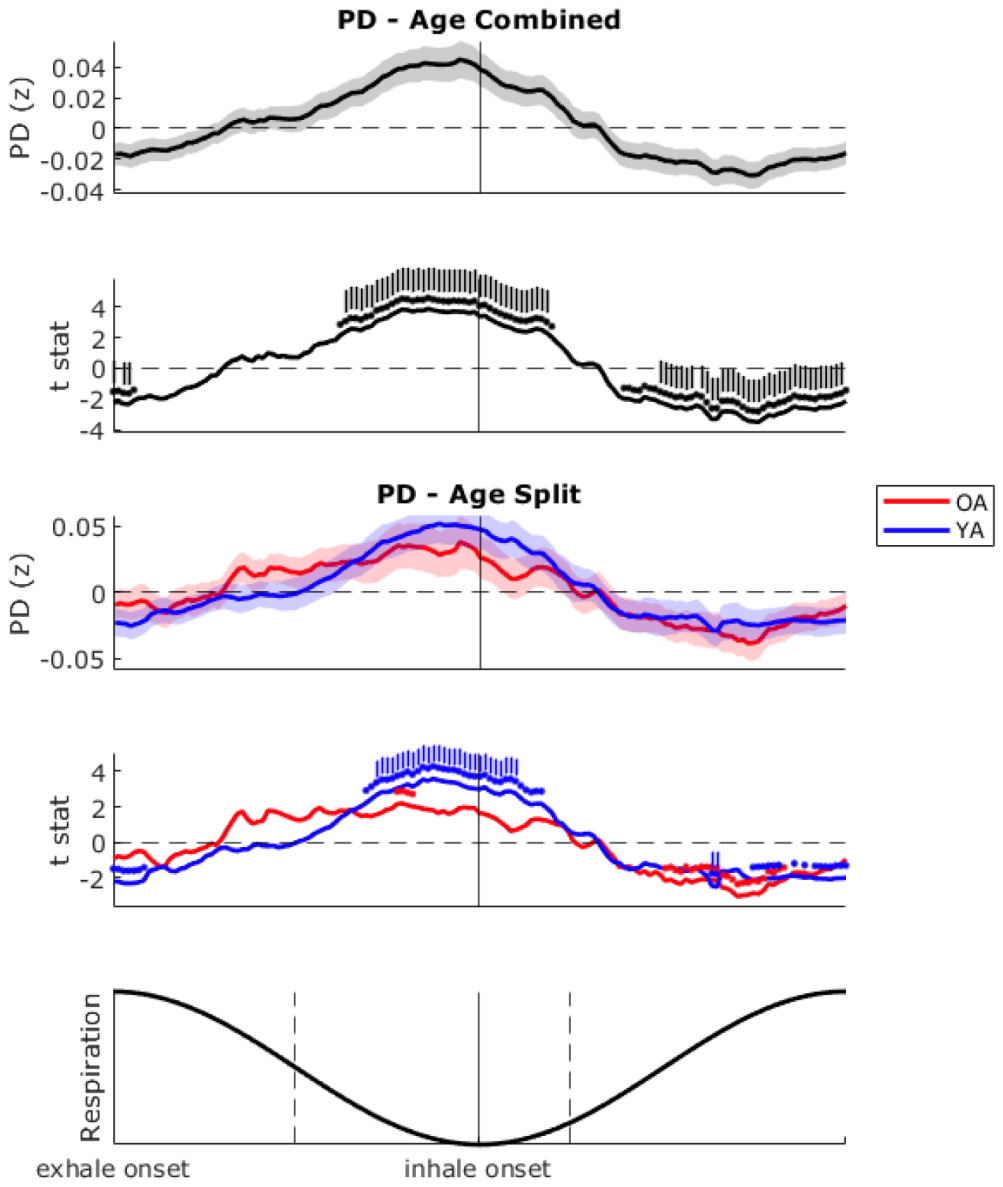
Pupil diameter amplitude, z-score normalised over the respiratory cycle. After preprocessing, pupil diameter was averaged within a moving phase window bin across the respiratory cycle, window of length π/36 with a 75% overlap. Plotted line with shaded area shows the mean normalised pupil diameter amplitude for each respiratory phase bin, shaded area represents the standard error of the mean (SEM). Plotted line with no shading shows the*t*statistic resulting from one-sample permutation testing against 1000 sets of simulation data. Dots above the line indicate significant (*p*< 0.05) phase bins and dashes indicate phases still significant following multiple comparisons correction. Top shows resultant plot, age groups combined (black), right shows split by the older adult (OA; red) and younger adult (YA; blue) groups. Bottom graphs show the respiratory waveform for reference. The dashed lines on the respiratory waveform represent an approximate window which was commonly phase-locked to task events (as per previous analyses).

Pupil diameter showed a smooth oscillation over the respiratory cycle (Fig. 5), peaking just prior to the exhalation-to-inhalation transition, and troughing just after it. When averaging across age groups, these phase deviations were significantly higher than chance (vs. 1000 surrogate values), and survived Benjamini–Hochberg correction (controls false discovery rate, FDR). For YA, these phase deviations also survived FDR corrections, but for the OA they did not.

EEG power over the respiratory cycle is plotted inFigure 6, and was also subject to significance testing and FDR correction. Frontal delta (1–4 Hz), theta (4–7 Hz), and posterior alpha (8–14 Hz) showed a similar modulation pattern over the respiratory cycle. Delta and theta power were significantly lower from peak inhalation through to late exhalation, whereas alpha power was lower only around the inhalation-to-exhalation transition. Delta, theta, and alpha were significantly higher in early inhalation. The posterior steady-state visually evoked potential (SSVEP; 25 Hz) was significantly lower over the exhalation-to-inhalation transition, and higher in late inhalation. Whilst similar patterns emerged for the OA and YA, the OA showed a more pronounced modulation for delta, theta, and alpha. YA, however, showed more modulation of the SSVEP. Out of the 143 phase bin windows, the OA had significant deviations from 0: 69.2% of delta, 69.9% theta, 35.0% alpha, and 0% SSVEP. YA: 12.6% delta, 6.9% theta, 0% alpha, and 26.6% SSVEP.

**Fig. 6.**
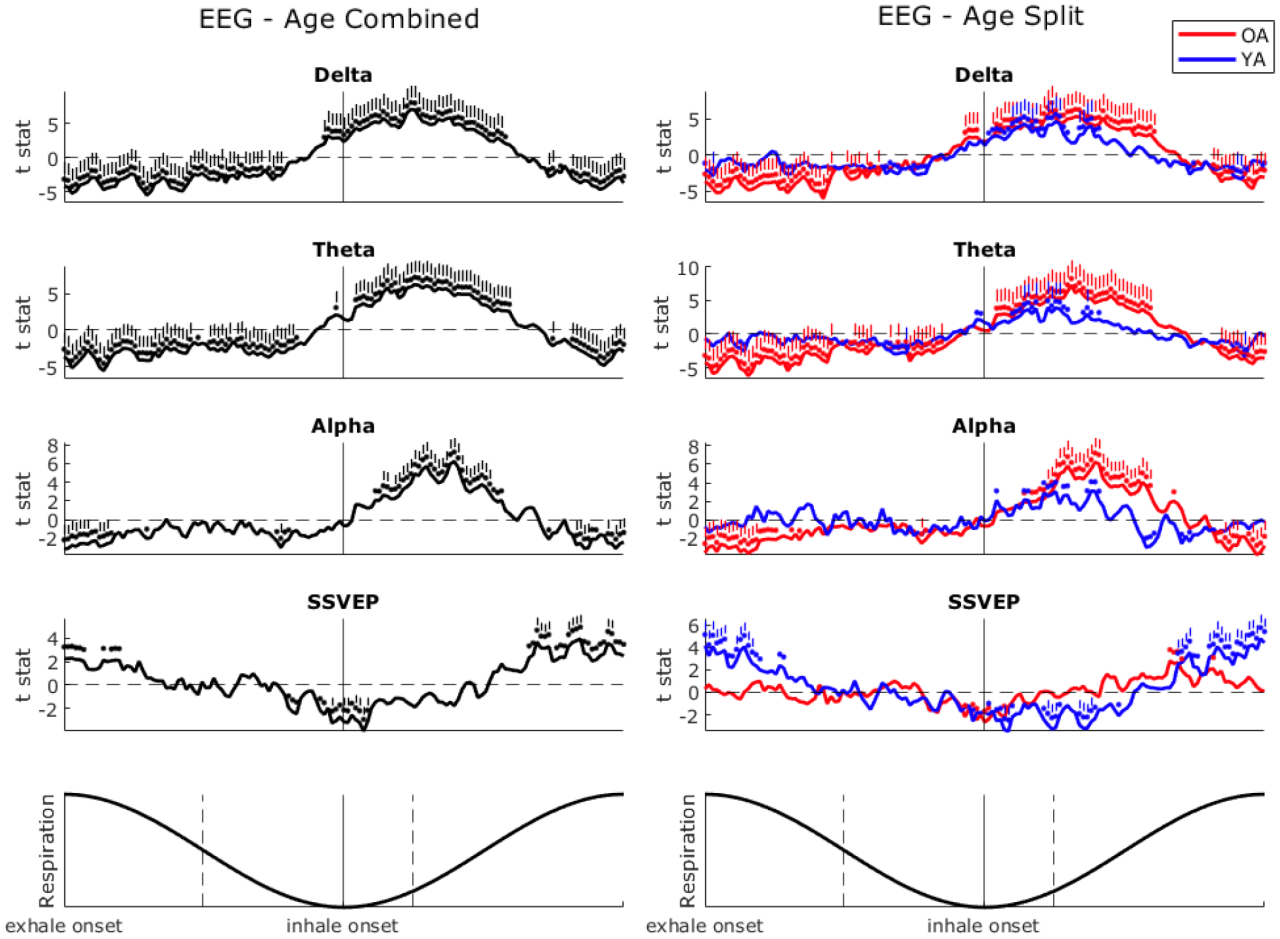
Electroencephalography (EEG)-derived frontal delta and theta, and posterior alpha and steady-state visually evoked potential (SSVEP) over the respiratory cycle. After preprocessing and organising by frequency, frequency-power was averaged within a moving phase window bin across the respiratory cycle, window of length π/36 with a 75% overlap. Plotted line shows the*t*statistic resulting from one-sample permutation testing against 1000 sets of simulation data. Dots above the line indicate significant (*p*< 0.05) phase bins and dashes indicate phases still significant following multiple comparisons correction. Left shows resultant plot, age groups combined (black), right shows split by the older adult (OA; red) and younger adult (YA; blue) groups. Bottom graphs show the respiratory waveform for reference. The dashed lines on the respiratory waveform represent an approximate window which was commonly phase-locked to task events (as per previous analyses).

## 4 Discussion

Here we observed consistent respiratory phase-locking behaviour so that late exhalation to early inhalation phases was synchronised with salient events in our sustained attention task. Further, there was evidence to suggest that respiratory dynamics were actively altered to accommodate the varying inter-target intervals, ensuring late exhalation phase-target synchronisation. Such anticipation of targets was also evident in peri-target trends in pupil diameter, indicating predictive preparation of the arousal system. This respiratory phase-locking behaviour was more pronounced in the older adult (OA) than in the younger adult (YA) group. Whilst these two age groups exhibited similar task performance, they differed in how consistently they maintained their attention to the task, evidenced by self-report, pupil diameter, and electroencephalographic indices of attention. We also showed that pupil diameter, frontal delta, theta, and posterior alpha and steady-state visually evoked potential (SSVEP), as indices of the arousal and attentional systems, were significantly modulated with respect to the target-synchronised respiratory phase window. The OA showed a more pronounced delta, theta, and alpha pattern, whereas the YA showed a more pronounced modulation of the SSVEP and pupil diameter. Collectively, we demonstrate that respiration can dynamically adapt to synchronise with changing inter-target intervals, more so in individuals with greater applied task attention. Respiratory phases synchronised with task events were characterised by low slow wave power and high pupil diameter, possibly reflecting a higher arousal, task-attuned state. The converse appeared to be the case in subsequent respiratory phases, possibly indicative of an attentional break. The degree of task-applied attention modulates this effect, however, in an index-dependant manner.

### 4.1 Older adults exhibited exploitative task attention

Our groups of YA and OA were intended to provide a natural division according to attentional strategies. The OA gave significantly more “focus” (vs. mind wandering) reports than the YA, consistent with the previous study to apply this task to these age groups (Moran, 2021;Moran et al., 2025) and a robust finding in this area of ageing research generally (Diede et al., 2022;Jackson & Balota, 2012;Maillet & Schacter, 2016;Robison et al., 2022;Seli et al., 2017;Welhaf et al., 2024). In complement to higher focus reports, and also consistent withMoran et al. (2021,^2025^), the OA showed differences in target-locked physiological indices which indicated a more “exploitative” attentional strategy than the YA. The OA appeared to encode the target evolution more reliably (steady-state visually evoked potential, SSVEP), show a stronger motor preparation pre-response (left hemisphere beta, LHB), exhibit lower variation in pre-target posterior alpha (visual processing and attention;Clayton et al., 2018), and demonstrate a greater evoked pupil diameter response to targets. The OA, therefore, appeared to show a more consistently vigilant state over the task. This contrasts with the YA, who exhibited signs of an “explorative” mode, with attentional indices indicating a more fluctuating task focus.

### 4.2 OA phase-lock respiration to the task more than YA and both groups show respiratory adaption

We extend these differences in attentional strategy to show that the OA also synchronised their respiration more strongly to the times of target response, thought probe (TP) presentation, and TP response. We also discovered evidence that this respiration-task event phase-locking involved an active respiratory adaption to ensure that targets and TP occurred in exhalation.

The strength of respiration-target phase-locking was similar, despite whether the target was after a 3, 5, or 7 s ITI. And although the phase-locked angle did shift slightly later in the respiratory cycle with increasing ITI, it remained within the late exhalation–early inhalation phase window. Additionally, the strength of phase-locking was related to more random variability in respiration rate. Collectively, these findings imply that respiration underwent some adaption to maintain a relatively consistent respiratory phase-locking, accommodating variable ITI. Our grand-average plots showing respiratory phase shifts over the target period (Fig. 4) show a robust target response involving an exhalation and subsequent inhalation, and otherwise fairly inconsistent phases changes in between responses. It is possible, that besides inter-trial and inter-individual variability, that this lack of phase change over the target presentation period also partially represents more constrained respiratory dynamics here. Since an exhalation response to the target was prioritised as a consistent phase shift, this would necessitate that there was any breath to exhale. The suggestion here is that respiratory dynamics are constrained between responding to one target and the next, in order to keep the breath within a range that would allow a certain magnitude of (exhalation) response. As evidence that participants had an implicit awareness of target expectancy, and, therefore, able to alter respiratory dynamics accordingly, we show pupil diameter dynamics over the target. Pupil diameter is indicative of moment-to-moment arousal, and was at its highest at the time of target presentation when the ITI was 3 s. If the ITI was longer, pupil diameter gradually declined until an evoked response. This indicates that the arousal system was primed ready to detect the target at the minimum ITI, reflecting anticipation of the target.

This active synchronisation between respiration and the timing of targets and TP implies some degree of rhythmic entrainment. Despite the variability in their periodicity of occurrence, the majority of participants appeared to consistently align certain respiratory phases with these times. This demonstrates an adaptive and dynamic process which could underlie anticipation of environmental rhythms. We further believe that our study contributes a unique demonstration of an anticipatory action of respiratory phase-locking due to the present task having both sudden-onset task events (TPs), which are typical of computer-based psychology tasks, and a stimulus which is presented continuously for multiple targets—the periodic gradual fades in contrast. This reported effect suggests an anticipatory action of respiratory phase-locking, in addition to reactive behaviours.

The respiratory phase-locking to the TP response is perhaps less appropriate to be termed “entrainment” as this event did not follow the rhythm imposed by the task as such. Instead, this result could be demonstrating strong respiratory-phase-locking to voluntary motor actions, in this case one that restarted the task following the TP. This result is highly consistent with that ofPerl et al. (2019)who also found that participants self-initiated task trials in early inhalation. This field of research should continue to “map” the respiratory cycle with regard to the different sensory, cognitive, and motor processes which show phase preference for a better understanding of what these phases have to offer the mental and neural dynamics.

Whilst respiratory-task phase-locking has been reported across many tasks (Grund et al., 2022;Huijbers et al., 2014;Johannknecht & Kayser, 2022;Melnychuk et al., 2018;Park et al., 2020;Perl et al., 2019), there is still little understanding as to the inter-individual variability of this effect. Although respiratory-task phase-locking was a majority finding in our participants, it was not ubiquitous. Further research is needed to elucidate which individuals show this tendency. We discovered a contributing factor of age group. Respiratory-task phase-locking did not significantly change over the hour of testing, nor within each block, and, therefore, participants appeared to learn the task structure rapidly, and maintain their phase-locking behaviour consistently. However, it is still uncertain whether respiratory phase-locking is dependent on the traits of an individual or the attentional state they enter the task in.

We propose that a tendency for vigilant (vs. wavering) attention increases respiratory-task phase-locking behaviour. We interpret our results under the assumption that the OA applied their cognitive resources more strongly to the task than the YA as they may have found the sustained attentional engagement demanding. We speculate that the OA could have utilised their respiratory rhythm as an additional and interoceptive attentional anchoring to aid stability in task engagement.

### 4.3 Physiological indices of arousal fluctuate over the respiratory cycle

Late exhalation to early inhalation appeared to be a crucial respiratory phase window for experiencing task events. Thus, we sought to characterise respiratory phases by their physiological background, specifically, the dynamics of pupil diameter and brain oscillations which are reflective of arousal and attentional state.

Pupil diameter was relatively high over late exhalation and early inhalation, aligning with the respiratory phase window most commonly synchronised with task events. Pupil diameter was relatively low over mid-to-late inhalation. Frontal delta, theta, and to some extent posterior alpha were relatively low prior to this phase-locked window and relatively high over early-to-mid inhalation.

Pupil diameter is a well-established peripheral marker of arousal, modulated by the autonomic nervous system through sympathetic and parasympathetic influences (Mm et al., 2008;Steinhauer et al., 2004). Larger pupil diameter is often associated with increased noradrenergic activity, heightened vigilance, and greater attentional engagement (Aston-Jones & Cohen, 2005;Reimer et al., 2016). The observed relative elevation in pupil diameter during late exhalation and early inhalation suggests a state of increased readiness to process external stimuli. Conversely, the relative reduction in pupil diameter during mid-to-late inhalation may reflect a transient shift towards a less aroused state. Considering our evidence that task targets are anticipated by the pupillary and respiratory systems, it is possible that this state reflects a transient disengagement from target anticipation.

Similarly, the power of EEG oscillations provides an insight into underlying neural dynamics governing arousal and attention. The observed modulation of frontal delta and theta and posterior alpha power aligns with their roles in regulating internal–external attentional control. Their relative suppression prior to the phase-locked window and subsequent increase during early-to-mid inhalation may indicate a dynamic shift from an anticipatory task-focused state to a disengaged mind wandering state. The steady-state visually evoked potential (SSVEP) was lowest over the exhalation-to-inhalation transition and highest over the inhalation-to-exhalation transition. The SSVEP tracks the flickering stimulus and decreases when the stimulus faded in contrast. Thus, it makes sense that it was lower over the target-expectancy period. However, it is also possible that this pattern reflects optimised and disrupted phases for sensory processing more generally across the respiratory cycle. Previous studies have shown that speeded responses (Saltafossi et al., 2025) and memory retrieval (Nakamura et al., 2018), and concurrent cortical activity (Nakamura et al., 2022) are disrupted by the exhalation-to-inhalation transition.

Together, these findings suggest that respiratory phase-dependent fluctuations in pupil diameter and EEG oscillations reflect systematic variations in arousal and attention. We speculate that the respiratory-dependant modulation patterns indicate an optimisation of attentional resources around the respiratory phases where task events were expected to occur, and then a facilitation of an attentional break from the task outside these phases.

The age-related differences to respiratory modulation patterns highlight how respiration differentially interacts with arousal, endogenous neural oscillations, and externally driven sensory responses. Whilst both YA and OA exhibited similar pupil diameter modulation across the respiratory cycle, this effect was significantly stronger in YA, suggesting that their arousal state was more dynamically coupled to respiration. At the neural level, OA showed significantly greater respiratory modulation of delta, theta, and alpha power, which may indicate a stronger reliance on respiration-linked oscillatory dynamics to support sustained attention. Given their overall greater task focus, this enhanced respiratory–brain coupling may reflect a compensatory mechanism for maintaining attentional stability. YA exhibited stronger respiratory modulation of the SSVEP, suggesting that their sensory processing was more directly influenced by respiration. The OA showed a more faithful tracking of the SSVEP as it gradually declined over the course of target evolution. (Supplementary Materials,Fig. S1b). This sustained tracking may have made their sensory processing less amenable to respiratory influence, as their attentional resources were more continuously engaged in following the external stimulus rather than fluctuating with respiratory phase.

In summary, the OA show stronger respiratory modulation of intrinsic attention-supporting oscillations, and YA display greater respiration modulation of arousal and sensory processing.

One interpretation of this pattern of results in totality is that the YA utilised cognitive flexibility to pay less continuous focus to the task, allowing respiratory-modulated fluctuations in their arousal and sensory processing to inform them of when to expect a target. Alternatively, the older adults’ strategy was to maintain intentional vigilance towards the task, as unintentional disengagement could be harder to recover from, with age-related handicaps in cognitive flexibility. Their arousal and sensory processing systems were slightly more stable to support this. However, they also have top-down modulated executive attention (and supporting oscillations) to be primed for periods of target expectancy, then, an intentional attentional recovery shortly after the target when another one is not yet expected. In other words, the younger adults tended to intentionally disengage in a more “automated” mode of respiratory-modulated attention possibly relying on bottom-up respiratory modulated signals, whereas older adults tended to intentionally and strategically engage and disengage with the task, guided by the respiratory rhythm and its coupling to top-down cortical signals.

We make a speculation from this: given respiratory modulation occurs globally across most cortical areas (Kluger & Gross, 2021), our findings might suggest that respiratory phase-locking alternates from more bottom-up obligatory processing of information (arousal/sensory fluctuations) when focus is low but locks to cortical low-frequency rhythms when focus is high. It would be interesting to experiment with an attention manipulation in this context, for example, applying the GradCCD-ES but instructing one group to passively observe and another group to give responses (as in the present study).

### 4.4 Other remarks

Late exhalation phases were the preferred period of the respiratory cycle for experiencing task targets. Why these specific phases? From the current data we can only speculate. For instance, we noted that indices of the arousal and attentional systems were seemingly optimised with respect to this window, however, the direction of causality is not clear here. It is possible that the arousal and attentional systems anticipate target detection and then respiration is phase-locked accordingly. Alternatively, perhaps respiration has a fixed phase–amplitude coupling relationship with pupil diameter and brain oscillations, and thus, the inherent conditions around late exhalation are most conducive for this type of target detection, and are applied accordingly. Which then offers another area of speculation: what are the relative contributions of the task context which determine the synchronised respiratory phase? Research has shown differential phase-locking to stimulus and response (Johannknecht & Kayser, 2022), so which is being prioritised here? Additionally, does the sensory domain influence which respiratory phase is locked? For instance, in a study on visual perception, early-to-mid inhalation appeared to be most conducive to task performance (Kluger et al., 2021), whereas a study on tactile perception showed a preference for early exhalation (Grund et al., 2022). In the latter study, it was concluded that the relative quiescence of the respiratory and cardiac systems in exhalation was facilitative of fine target detection. It is possible that this same interpretation applies to our present study, and that late exhalation is a phase of higher quiescence and less interference with sensory detection systems. As demonstrated byKluger et al. (2023)in a separate study, the respiratory-cyclic modulation of the neural excitation–inhibition balance may play a crucial role in determining the optimal state for a specific sensory-cognitive or motor context.

It is worth discussing how the present pattern for pupil diameter modulation over the respiratory cycle compares with that which has recently been reported. Following a review which concluded that evidence for a respiratory-phase modulation of pupil diameter was mixed (Schaefer et al., 2023),Schaefer et al. (2024)demonstrated a clear effect during both resting and task conditions. They found that peak pupil diameter occurred over early exhalation during their task. Our pattern is not so dissimilar, with peak pupil diameter occurring over late exhalation, however, it is slightly shifted in phase. It is intriguing to speculate that the difference in task dynamics and thus possible differences in the timing of target anticipation could influence the respiratory–pupillary coupling. For example, whilst the authors also applied a visual perception task, their trials were sudden-onset in nature and the ITI was considerably shorter (1200–3500 ms). A recent paper from our own research group also showed a pupillary peak at early exhalation during another sustained attention task (Andrews et al., 2025), more aligned in pattern to that ofSchaefer et al. (2024). There may be something distinctive about the dynamics of the GradCCD-ES task that produced this patten. Future research utilising a variety of tasks and manipulating ITI should prove insightful on this point.

Our findings of a link between respiratory and pupillary dynamics are consistent with a dynamical systems model in which respiratory and cortical attentional inputs to the locus coeruleus can mediate the stability of the prevailing attentional state (Melnychuk et al., 2018). Previous research by this group showed significant synchronisation and information transfer (Granger causality) between respiration, pupil diameter, and frontal EEG during rest (Melnychuk et al., 2021). Our work provides additional evidence for this model by showing that pupil diameter and frontal EEG are modulated across the respiratory cycle during a sustained attention task. Importantly, the nature of the coupling appears to be dependent on the strength of top-down applied attention to a task and the degree of respiration-task phase-locking. These may be fruitful parameters to experiment with in the future to better understand how respiration and attention interact with each other. For example, one could note the effects on respiratory-pupillary phase-coupling when altering respiratory-task phase-locking though changing ITI or when altering attentional demand through changing the difficulty of the task.

### 4.5 Limitations

A general and important limitation to this study is using age to divide participants according to attentional engagement. These groups differ in several other ways, such as motivational factors, technological familiarity, and of high relevance, changes in neuromodulatory systems, particularly reductions in locus coeruleus (LC) integrity, and norepinephrine availability in OA (Mather & Harley, 2016). The OA required a passing score on a standardised test of cognitive abilities and all task metrics except self-reported focus were comparable with the YA. Thus, although an oversimplification, utilising these groups for the present purposes was likely appropriate. Future work should consider ways to determine a contribution of top-down attention on pupillary and respiratory activity with less confounding factors. For example, a group similar in age could complete this paradigm with differing attentional demands, by, for example, giving different task instructions or manipulating the degree of stimulus contrast change. Additionally, for example, respiratory phase-locking strength could be correlated to performance in other standardised sustained attention tasks such as the sustained attention to response task (SART).

Whilst respiration is shown to modulate attention and cognitive performance, the directionality and underlying mechanisms remain complex. It is unclear whether respiration directly influences attention or whether both are co-regulated by a common neuromodulatory system (e.g., LC-noradrenaline). Future studies could employ experimental manipulations of breathing (e.g., paced breathing) to clarify causality. Preliminary findings from our own study suggests that paced breathing does support attentional anchoring (Andrews et al., 2025)

We suggest here that applied task attention is a contributing factor to respiratory phase-locking behaviour, however, additional factors are required to fully explain why some individuals show this and others do not. For example, it could be that individual differences in interoceptive awareness, the ability to consciously perceive internal bodily signals including respiration, play a role in respiratory phase-locking behaviour. Individuals with higher interoceptive awareness may be more sensitive to subtle respiratory cues and could unconsciously synchronise cognitive processing with their breathing cycle to optimise performance. Conversely, those with lower interoceptive awareness may exhibit weaker phase-locking due to a reduced ability to detect and align cognitive processes with respiratory rhythms. Future research should explore how interoceptive sensitivity interacts with attentional engagement and respiratory dynamics, potentially using measures such as heartbeat detection tasks (Kleckner et al., 2015), or respiratory interoceptive tasks (Nikolova et al., 2022) or self-report questionnaires to quantify interoceptive awareness (Mehling et al., 2018).

### 4.6 Conclusion

This study provides compelling evidence that respiration adaptively phase-locks to salient task events, with stronger synchronisation observed in individuals exhibiting greater task-focused attention. Our findings suggest that this phase-locking behaviour is not merely reactive but represents an anticipatory adaptation of respiratory dynamics, particularly in response to varying inter-target intervals. Notably, older adults demonstrated a more pronounced tendency for respiratory phase-locking, which we interpret as an attentional strategy aimed at sustaining cognitive engagement. This aligns with broader differences in attentional strategies, wherein older adults maintained a more vigilant and exploitative task focus, whilst younger adults exhibited a more flexible and explorative approach.

Physiological markers of arousal, including pupil diameter and EEG oscillations, systematically fluctuated over the respiratory cycle, with late exhalation to early inhalation emerging as a crucial window for task engagement. This phase corresponded to heightened arousal and attentional readiness, as indicated by increased pupil diameter and modulations in delta, theta, and alpha oscillatory activity. The differential respiratory modulation of neural and physiological indices between younger and older adults further underscores the role of respiration in shaping age-related attentional dynamics.

Importantly, these findings contribute to a growing body of research highlighting the intricate relationship between respiration, cognition, and neural function. By demonstrating that respiration can act as a dynamic scaffold for attention, our study suggests that respiratory rhythms may serve as a modifiable target for enhancing cognitive performance, particularly in contexts requiring sustained vigilance. Future research should explore the inter-individual variability in respiratory phase-locking and its potential as a biomarker for attentional control, as well as investigate whether training interventions can leverage respiratory dynamics to optimise cognitive and neural functioning across the lifespan.

## Supplementary Material

Supplementary Material

## Data Availability

Data and code available upon request.

## References

[IMAG.a.26-b1] Andrews , R. , Melnychuk , M. , Moran , S. , Walsh , T. , Boylan , S. , & Dockree , P. ( 2025 ). Paced breathing associated with pupil diameter oscillations at the same rate and reduced lapses in attention . Psychophysiology , 62 ( 2 ), e70003 . 10.1111/psyp.70003 39905564 PMC11794674

[IMAG.a.26-b2] Aston-Jones , G. , & Cohen , J. D. ( 2005 ). An integrative theory of locus coeruleus-norepinephrine function: Adaptive gain and optimal performance . Annual Review of Neuroscience , 28 , 403 – 450 . 10.1146/annurev.neuro.28.061604.135709 16022602

[IMAG.a.26-b3] Bang , D. , Luo , Y. , Barbosa , L. S. , Batten , S. R. , Hadj-Amar , B. , Twomey , T. , Melville , N. , White , J. P. , Torres , A. , Celaya , X. , Ramaiah , P. , McClure , S. M. , Brewer , G. A. , Bina , R. W. , Lohrenz , T. , Casas , B. , Chiu , P. H. , Vannucci , M. , Kishida , K. T. , … Montague , P. R. ( 2023 ). Noradrenaline tracks emotional modulation of attention in human amygdala . Current Biology , 33 ( 22 ), 5003.e6 – 5010.e6 . 10.1016/j.cub.2023.09.074 37875110 PMC10957395

[IMAG.a.26-b4] Benjamini , Y. , & Hochberg , Y. ( 1995 ). Controlling the false discovery rate: A practical and powerful approach to multiple testing . Journal of the Royal Statistical Society. Series B (Methodological) , 57 ( 1 ), 289 – 300 . 10.1111/j.2517-6161.1995.tb02031.x

[IMAG.a.26-b5] Berens , P. ( 2009 ). CircStat: A MATLAB Toolbox for Circular Statistics . Journal of Statistical Software , 31 , 1 – 21 . 10.18637/jss.v031.i10

[IMAG.a.26-b6] Brainard , D. H. ( 1997 ). The psychophysics toolbox . Spatial Vision , 10 ( 4 ), 433 – 436 . 10.1163/156856897X00357 9176952

[IMAG.a.26-b7] Clayton , M. S. , Yeung , N. , & Cohen Kadosh , R . ( 2018 ). The many characters of visual alpha oscillations . European Journal of Neuroscience , 48 ( 7 ), 2498 – 2508 . 10.1111/ejn.13747 29044823

[IMAG.a.26-b8] Cremers , J. , & Klugkist , I. ( 2018 ). One direction? A tutorial for circular data analysis using R with examples in cognitive psychology . Frontiers in Psychology , *9* , 2040. https://www.frontiersin.org/articles/10.3389/fpsyg.2018.02040 10.3389/fpsyg.2018.02040 PMC6218623 30425670

[IMAG.a.26-b9] Diede , N. T. , Gyurkovics , M. , Nicosia , J. , Diede , A. , & Bugg , J. M. ( 2022 ). The effect of context on mind-wandering in younger and older adults . Consciousness and Cognition , 97 , 103256 . 10.1016/j.concog.2021.103256 34902670

[IMAG.a.26-b10] Elman , J. A. , Panizzon , M. S. , Hagler , D. J. , Eyler , L. T. , Granholm , E. L. , Fennema-Notestine , C. , Lyons , M. J. , McEvoy , L. K. , Franz , C. E. , Dale , A. M. , & Kremen , W. S. ( 2017 ). Task-evoked pupil dilation and BOLD variance as indicators of locus coeruleus dysfunction . Cortex; a Journal Devoted to the Study of the Nervous System and Behavior , 97 , 60 – 69 . 10.1016/j.cortex.2017.09.025 29096196 PMC5716879

[IMAG.a.26-b11] Grund , M. , Al , E. , Pabst , M. , Dabbagh , A. , Stephani , T. , Nierhaus , T. , Gaebler , M. , & Villringer , A. ( 2022 ). Respiration, heartbeat, and conscious tactile perception . The Journal of Neuroscience: The Official Journal of the Society for Neuroscience , 42 ( 4 ), 643 – 656 . 10.1523/JNEUROSCI.0592-21.2021 34853084 PMC8805629

[IMAG.a.26-b12] Heck , D. H. , Kozma , R. , & Kay , L. M. ( 2019 ). The rhythm of memory: How breathing shapes memory function . Journal of Neurophysiology , 122 ( 2 ), 563 – 571 . 10.1152/jn.00200.2019 31215344 PMC6734396

[IMAG.a.26-b13] Heck , D. H. , McAfee , S. S. , Liu , Y. , Babajani-Feremi , A. , Rezaie , R. , Freeman , W. J. , Wheless , J. W. , Papanicolaou , A. C. , Ruszinkó , M. , Sokolov , Y. , & Kozma , R. ( 2017 ). Breathing as a fundamental rhythm of brain function . Frontiers in Neural Circuits , 10 , 115 . https://www.frontiersin.org/articles/10.3389/fncir.2016.00115 28127277 10.3389/fncir.2016.00115 PMC5226946

[IMAG.a.26-b14] Henderson , R. R. , Bradley , M. M. , & Lang , P. J. ( 2018 ). Emotional imagery and pupil diameter . Psychophysiology , 55 ( 6 ), e13050 . 10.1111/psyp.13050 29266253 PMC5940515

[IMAG.a.26-b15] Huijbers , W. , Pennartz , C. M. A. , Beldzik , E. , Domagalik , A. , Vinck , M. , Hofman , W. F. , Cabeza , R. , & Daselaar , S. M. ( 2014 ). Respiration phase-locks to fast stimulus presentations: Implications for the interpretation of posterior midline ‘deactivations’ . Human Brain Mapping , 35 ( 9 ), 4932 – 4943 . 10.1002/hbm.22523 24737724 PMC4445359

[IMAG.a.26-b16] Jackson , J. D. , & Balota , D. A. ( 2012 ). Mind-wandering in younger and older adults: Converging evidence from the sustained attention to response task and reading for comprehension . Psychology and Aging , 27 ( 1 ), 106 – 119 . 10.1037/a0023933 21707183 PMC3508668

[IMAG.a.26-b17] Johannknecht , M. , & Kayser , C. ( 2022 ). The influence of the respiratory cycle on reaction times in sensory-cognitive paradigms . Scientific Reports , 12 ( 1 ), 2586 . 10.1038/s41598-022-06364-8 35173204 PMC8850565

[IMAG.a.26-b18] Jordão , M. , Ferreira-Santos , F. , Pinho , M. S. , & St. Jacques , P. L . ( 2019 ). Meta-analysis of aging effects in mind wandering: Methodological and sociodemographic factors . Psychology and Aging , 34 ( 4 ), 531 – 544 . 10.1037/pag0000356 31033303

[IMAG.a.26-b19] Joshi , S. , Li , Y. , Kalwani , R. M. , & Gold , J. I. ( 2016 ). Relationships between pupil diameter and neuronal activity in the locus coeruleus, colliculi, and cingulate cortex . Neuron , 89 ( 1 ), 221 – 234 . 10.1016/j.neuron.2015.11.028 26711118 PMC4707070

[IMAG.a.26-b20] Kam , J. W. Y. , Rahnuma , T. , Park , Y. E. , & Hart , C. M. ( 2022 ). Electrophysiological markers of mind wandering: A systematic review . NeuroImage , 258 , 119372 . 10.1016/j.neuroimage.2022.119372 35700946

[IMAG.a.26-b21] Kayser , J. , & Tenke , C. E. ( 2015 ). On the benefits of using surface Laplacian (Current Source Density) methodology in electrophysiology . International Journal of Psychophysiology: Official Journal of the International Organization of Psychophysiology , 97 ( 3 ), 171 – 173 . 10.1016/j.ijpsycho.2015.06.001 26071227 PMC4610715

[IMAG.a.26-b22] Kilavik , B. E. , Zaepffel , M. , Brovelli , A. , MacKay , W. A. , & Riehle , A. ( 2013 ). The ups and downs of β oscillations in sensorimotor cortex . Experimental Neurology , 245 , 15 – 26 . 10.1016/j.expneurol.2012.09.014 23022918

[IMAG.a.26-b23] Kleckner , I. R. , Wormwood , J. B. , Simmons , W. K. , Barrett1 , L. F. , & Quigley , K. S. ( 2015 ). Methodological recommendations for a heartbeat detection-based measure of interoceptive sensitivity . Psychophysiology , 52 ( 11 ), 1432 – 1440 . 10.1111/psyp.12503 26265009 PMC4821012

[IMAG.a.26-b24] Kluger , D. S. , Balestrieri , E. , Busch , N. A. , & Gross , J. ( 2021 ). Respiration aligns perception with neural excitability . eLife , 10 , e70907 . 10.7554/eLife.70907 34904567 PMC8763394

[IMAG.a.26-b25] Kluger , D. S. , Forster , C. , Abbasi , O. , Chalas , N. , Villringer , A. , & Gross , J. ( 2023 ). Modulatory dynamics of periodic and aperiodic activity in respiration-brain coupling . Nature Communications , 14 ( 1 ), 4699 . 10.1038/s41467-023-40250-9 PMC10404236 37543697

[IMAG.a.26-b26] Kluger , D. S. , & Gross , J. ( 2021 ). Respiration modulates oscillatory neural network activity at rest . PLoS Biology , 19 ( 11 ), e3001457 . 10.1371/journal.pbio.3001457 34762645 PMC8610250

[IMAG.a.26-b27] Kluger , D. S. , Gross , J. , & Keitel , C. ( 2023 ). A dynamic link between respiration and arousal . bioRxiv . 10.1101/2023.10.06.561178 PMC11580776 39379154

[IMAG.a.26-b28] Lindenberger , U. , & Mayr , U. ( 2014 ). Cognitive aging: Is there a dark side to environmental support? Trends in Cognitive Sciences , 18 ( 1 ), 7 – 15 . 10.1016/j.tics.2013.10.006 24210962 PMC3969029

[IMAG.a.26-b29] Loughnane , G. M. , Brosnan , M. B. , Barnes , J. J. M. , Dean , A. , Nandam , S. L. , O’Connell , R. G. , & Bellgrove , M. A. ( 2019 ). Catecholamine modulation of evidence accumulation during perceptual decision formation: A randomized trial . Journal of Cognitive Neuroscience , 31 ( 7 ), 1044 – 1053 . 10.1162/jocn_a_01393 30883291

[IMAG.a.26-b30] Loughnane , G. M. , Newman , D. P. , Bellgrove , M. A. , Lalor , E. C. , Kelly , S. P. , & O’Connell , R. G. ( 2016 ). Target selection signals influence perceptual decisions by modulating the onset and rate of evidence accumulation . Current Biology , 26 ( 4 ), 496 – 502 . 10.1016/j.cub.2015.12.049 26853360

[IMAG.a.26-b31] Maillet , D. , & Schacter , D. L. ( 2016 ). From mind wandering to involuntary retrieval: Age-related differences in spontaneous cognitive processes . Neuropsychologia , 80 , 142 – 156 . 10.1016/j.neuropsychologia.2015.11.017 26617263 PMC4698179

[IMAG.a.26-b63] Mather , M. , & Harley , C. W. ( 2016 ). The locus coeruleus: Essential for maintaining cognitive function and the aging brain . Trends in Cognitive Sciences , 20 ( 3 ), 214 – 226 . https://doi.org/10.1016/j.tics.2016.01.001 26895736 10.1016/j.tics.2016.01.001 PMC4761411

[IMAG.a.26-b32] Mathôt , S. , Fabius , J. , Heusden , E. V. , & Stigchel , S. V. der. ( 2018 ). Safe and sensible preprocessing and baseline correction of pupil-size data . Behavior Research Methods , 50 ( 1 ), 94 . 10.3758/s13428-017-1007-2 29330763 PMC5809553

[IMAG.a.26-b33] McGovern , D. P. , Hayes , A. , Kelly , S. P. , & O’Connell , R. G. ( 2018 ). Reconciling age-related changes in behavioural and neural indices of human perceptual decision-making . Nature Human Behaviour , 2 ( 12 ), Article 12. 10.1038/s41562-018-0465-6 30988441

[IMAG.a.26-b34] Mehling , W. E. , Acree , M. , Stewart , A. , Silas , J. , & Jones , A. ( 2018 ). The Multidimensional Assessment of Interoceptive Awareness, Version 2 (MAIA-2) . PLoS One , 13 ( 12 ), e0208034 . 10.1371/journal.pone.0208034 30513087 PMC6279042

[IMAG.a.26-b35] Meissner , S. N. , Bächinger , M. , Kikkert , S. , Imhof , J. , Missura , S. , Carro Dominguez , M. , & Wenderoth , N. ( 2024 ). Self-regulating arousal via pupil-based biofeedback . Nature Human Behaviour , 8 , 43 – 62 . 10.1038/s41562-023-01729-z PMC10810759 37904022

[IMAG.a.26-b36] Melnychuk , M. C. , Dockree , P. M. , O’Connell , R. G. , Murphy , P. R. , Balsters , J. H. , & Robertson , I. H. ( 2018 ). Coupling of respiration and attention via the locus coeruleus: Effects of meditation and pranayama . Psychophysiology , 55 ( 9 ), e13091 . 10.1111/psyp.13091 29682753

[IMAG.a.26-b37] Melnychuk , M. C. , Robertson , I. H. , Plini , E. R. G. , & Dockree , P. M. ( 2021 ). A bridge between the breath and the brain: Synchronization of respiration, a pupillometric marker of the locus coeruleus, and an EEG marker of attentional control state . Brain Sciences , 11 ( 10 ), 1324 . 10.3390/brainsci11101324 34679389 PMC8534189

[IMAG.a.26-b38] Mm , B. , L , M. , Ma , E. , & Pj , L. ( 2008 ). The pupil as a measure of emotional arousal and autonomic activation . Psychophysiology , 45 ( 4 ), 602 – 607 . 10.1111/j.1469-8986.2008.00654.x 18282202 PMC3612940

[IMAG.a.26-b39] Moran , C. N. ( 2021 ). The Neuropsychological and Neurophysiological Signatures of Age-Related Differences in Mind-Wandering [Thesis, Trinity College Dublin. School of Psychology. Discipline of Psychology]. http://www.tara.tcd.ie/handle/2262/96805

[IMAG.a.26-b40] Moran , C. N. , McGovern , D. P. , Melnychuk , M. C. , Smeaton , A. , & Dockree , P. M. ( 2025 ). Oscillations of the wandering mind: Neural evidence for distinct exploration/exploitation strategies in younger and older adults . Human Brain Mapping , 46 ( 6 ), e70174 . 10.1002/hbm.70174 40287841 PMC12034160

[IMAG.a.26-b41] Moran , C. N. , McGovern , D. P. , Warren , G. , Grálaigh , R. Ó. , Kenney , J. P. M. , Smeaton , A. , & Dockree , P. M. ( 2021 ). Young and restless, old and focused: Age-differences in mind-wandering frequency and phenomenology . Psychology and Aging , 36 ( 2 ), 252 – 267 . 10.1037/pag0000526 33539151

[IMAG.a.26-b42] Murphy , P. R. , O’Connell , R. G. , O’Sullivan , M. , Robertson , I. H. , & Balsters , J. H. ( 2014 ). Pupil diameter covaries with BOLD activity in human locus coeruleus . Human Brain Mapping , 35 ( 8 ), 4140 – 4154 . 10.1002/hbm.22466 24510607 PMC6869043

[IMAG.a.26-b43] Nakamura , N. H. , Fukunaga , M. , & Oku , Y. ( 2018 ). Respiratory modulation of cognitive performance during the retrieval process . PLoS One , 13 ( 9 ), e0204021 . 10.1371/journal.pone.0204021 30216372 PMC6138381

[IMAG.a.26-b44] Nakamura , N. H. , Fukunaga , M. , Yamamoto , T. , Sadato , N. , & Oku , Y. ( 2022 ). Respiration-timing-dependent changes in activation of neural substrates during cognitive processes . Cerebral Cortex Communications , 3 ( 4 ), tgac038 . 10.1093/texcom/tgac038 36237849 PMC9552779

[IMAG.a.26-b45] Nasreddine , Z. S. , Phillips , N. A. , Bédirian , V. , Charbonneau , S. , Whitehead , V. , Collin , I. , Cummings , J. L. , & Chertkow , H. ( 2005 ). The Montreal Cognitive Assessment, MoCA: A brief screening tool for mild cognitive impairment . Journal of the American Geriatrics Society , 53 ( 4 ), 695 – 699 . 10.1111/j.1532-5415.2005.53221.x 15817019

[IMAG.a.26-b46] Nikolova , N. , Harrison , O. , Toohey , S. , Brændholt , M. , Legrand , N. , Correa , C. , Vejlø , M. , Jensen , M. S. , Fardo , F. , & Allen , M. ( 2022 ). The respiratory resistance sensitivity task: An automated method for quantifying respiratory interoception and metacognition . Biological Psychology , 170 , 108325 . 10.1016/j.biopsycho.2022.108325 35358604

[IMAG.a.26-b47] Norcia , A. M. , Appelbaum , L. G. , Ales , J. M. , Cottereau , B. R. , & Rossion , B. ( 2015 ). The steady-state visual evoked potential in vision research: A review . Journal of Vision , 15 ( 6 ), 4 . 10.1167/15.6.4 PMC4581566 26024451

[IMAG.a.26-b48] O’Connell , R. G. , Dockree , P. M. , & Kelly , S. P. ( 2012 ). A supramodal accumulation-to-bound signal that determines perceptual decisions in humans . Nature Neuroscience , 15 ( 12 ), 1729 – 1735 . 10.1038/nn.3248 23103963

[IMAG.a.26-b49] Oostenveld , R. , Fries , P. , Maris , E. , & Schoffelen , J.-M. ( 2010 ). FieldTrip: Open source software for advanced analysis of MEG, EEG, and invasive electrophysiological data . Computational Intelligence and Neuroscience , 2011 , e156869 . 10.1155/2011/156869 PMC3021840 21253357

[IMAG.a.26-b50] Park , H.-D. , Barnoud , C. , Trang , H. , Kannape , O. A. , Schaller , K. , & Blanke , O. ( 2020 ). Breathing is coupled with voluntary action and the cortical readiness potential . Nature Communications , 11 ( 1 ), 289 . 10.1038/s41467-019-13967-9 PMC7005287 32029711

[IMAG.a.26-b51] Perl , O. , Ravia , A. , Rubinson , M. , Eisen , A. , Soroka , T. , Mor , N. , Secundo , L. , & Sobel , N. ( 2019 ). Human non-olfactory cognition phase-locked with inhalation . Nature Human Behaviour , 3 ( 5 ), Article 5. 10.1038/s41562-019-0556-z 31089297

[IMAG.a.26-b52] Reimer , J. , McGinley , M. J. , Liu , Y. , Rodenkirch , C. , Wang , Q. , McCormick , D. A. , & Tolias , A. S. ( 2016 ). Pupil fluctuations track rapid changes in adrenergic and cholinergic activity in cortex . Nature Communications , 7 , 13289 . 10.1038/ncomms13289 PMC5105162 27824036

[IMAG.a.26-b53] Robison , M. K. , Diede , N. T. , Nicosia , J. , Ball , B. H. , & Bugg , J. M. ( 2022 ). A multimodal analysis of sustained attention in younger and older adults . Psychology and Aging , 37 ( 3 ), 307 – 325 . 10.1037/pag0000687 35446084 PMC10128103

[IMAG.a.26-b54] Saltafossi , M. , Zaccaro , A. , Kluger , D. S. , Perrucci , M. G. , Ferri , F. , & Costantini , M. ( 2025 ). Respiration facilitates behaviour during multisensory integration . bioRxiv . 10.1101/2025.01.10.632352 PMC12464358 40999648

[IMAG.a.26-b55] Schaefer , M. , Edwards , S. , Nordén , F. , Lundström , J. N. , & Arshamian , A. ( 2023 ). Inconclusive evidence that breathing shapes pupil dynamics in humans: A systematic review . Pflügers Archiv—European Journal of Physiology , 475 ( 1 ), 119 – 137 . 10.1007/s00424-022-02729-0 35871662 PMC9816272

[IMAG.a.26-b56] Schaefer , M. , Mathôt , S. , Lundqvist , M. , Lundström , J. N. , & Arshamian , A. ( 2024 ). The Respiratory-Pupillary Phase Effect: Pupils size is smallest around inhalation onset and largest during exhalation . bioRxiv . 10.1101/2024.06.27.599713 PMC11908488 39981599

[IMAG.a.26-b57] Seli , P. , Cheyne , J. A. , Xu , M. , Purdon , C. , & Smilek , D. ( 2015 ). Motivation, intentionality, and mind wandering: Implications for assessments of task-unrelated thought . Journal of Experimental Psychology: Learning, Memory, and Cognition , 41 ( 5 ), 1417 – 1425 . 10.1037/xlm0000116 25730306

[IMAG.a.26-b58] Seli , P. , Maillet , D. , Smilek , D. , Oakman , J. M. , & Schacter , D. L. ( 2017 ). Cognitive aging and the distinction between intentional and unintentional mind wandering . Psychology and Aging , 32 ( 4 ), 315 – 324 . 10.1037/pag0000172 28471215 PMC5459659

[IMAG.a.26-b59] Steinhauer , S. R. , Siegle , G. J. , Condray , R. , & Pless , M. ( 2004 ). Sympathetic and parasympathetic innervation of pupillary dilation during sustained processing . International Journal of Psychophysiology , 52 ( 1 ), 77 – 86 . 10.1016/j.ijpsycho.2003.12.005 15003374

[IMAG.a.26-b60] Vlemincx , E. , Van Diest , I. , & Van den Bergh , O. ( 2012 ). A sigh following sustained attention and mental stress: Effects on respiratory variability . Physiology & Behavior , 107 ( 1 ), 1 – 6 . 10.1016/j.physbeh.2012.05.013 22634279

[IMAG.a.26-b61] Wang , C.-A. , Baird , T. , Huang , J. , Coutinho , J. D. , Brien , D. C. , & Munoz , D. P. ( 2018 ). Arousal effects on pupil size, heart rate, and skin conductance in an emotional face task . Frontiers in Neurology , 9 , 1029 . 10.3389/fneur.2018.01029 30559707 PMC6287044

[IMAG.a.26-b62] Welhaf , M. S. , Banks , J. B. , & Bugg , J. M. ( 2024 ). Age-related differences in mind wandering: The role of emotional valence . The Journals of Gerontology. Series B, Psychological Sciences and Social Sciences , 79 ( 1 ), gbad151 . 10.1093/geronb/gbad151 37813376 PMC10745276

