# Supplementary material for: Arousal and sustained attention fluctuate differently with respiration in younger and older adults
Source: Imaging Neurosci (Camb). 2025 Jun 3;3:IMAG.a.26. doi: 10.1162/IMAG.a.26 (PMC12319817; doi:10.1162/IMAG.a.26)
Supplement: Supplementary Material [file imag.a.26_supp.pdf]

Supplemental Materials

Age group differences in behavioural, experiential, and physiological signatures of attention

Behaviour and Experience Sampling

In response to TPs, OA on average indicated 'Focused' 13.27 ± 0.65 / 16 times, versus reporting 'mind wandering', compared to YA who indicated 'Focused', which was significantly higher: $t(70) = 4.83$, $p < 0.001$, $d = 1.14$. Otherwise, task performance-related behavioural variables were not significantly different between the groups (Table S1). These findings echoed Moran's (2021, 2025, in press), except for their additional finding of OA having significantly lower RT CoV.

Target-Locked Neural Indices

Target-locked neural indices are plotted in Figure S1. Testing for differences between the age groups in parameters of the indices, the OA showed: a significantly more negative post-target SSVEP slope, $M = -0.003 \pm 0.0006$, than YA, $M = -0.0003 \pm 0.0007$, $t(60) = -2.48$, $p = 0.016$, $d = 0.27$; a significantly lower near-response LHB slope, OA $M = -0.002 \pm -0.0008$, YA $M = -0.001 \pm 0.0008$, $t(60) = -2.86$, $p = 0.006$, $d = -0.73$; and a significantly lower pre-target alpha power CoV, OA $M = 0.24 \pm 0.01$, YA $M = 0.29 \pm 0.02$, $U = 327$, $p = 0.03$, $r = -0.32$ (Table S2). These findings echoed Moran's (2021).

Behaviour and Experience Sampling

Independent Samples T-Test

	t	df	*p*	Cohen's *d*
RTm	1.227	70	0.224	0.290
RT CoV	-1.096	70	0.277	-0.259
HR	-0.465	70	0.644	-0.110
FA	1.377	70	0.173	0.325
**Focus (MW)**	(-)4.826	70	**< 0.001**	1.139

Table S1 – Results from means comparison tests between the age groups, OA and YA, testing for differences in task performance and experience sampling focus. The two groups did not significantly differ ($p > 0.05$) with regards to task performance metrics, but the OA did report significantly higher ($p < 0.001$) 'Focus' rather than 'Mind Wandering' (MW), in response to thought probes (TPs). FA = False Alarms.

Target-Locked Neural Indices Parameters

Independent Samples T-Test

	T	**df**	***p***	**Cohen's *d***
CPP Peak Amp	-1.535	60	0.130	-0.391
CPP Peak Latency, ms	0.396	60	0.693	0.101
CPP Slope	-0.787	60	0.434	-0.200
SSVEP Mean Amp[a]	-1.593	50.2	0.117	-0.398
**SSVEP Slope**	-2.476	60	**0.016**	-0.630
LHB Mean Amp	-1.444	60	0.154	-0.368
LHB Slope	-0.833	60	0.408	-0.212
LHB Trough	-1.679	60	0.098	-0.427
**LHB Slope (Response)**	-2.861	60	**0.006**	-0.728

Mann Whitney-U Test

	U	***p***	**Rank-Biserial Correlation**
**Alpha CoV**	327	**0.032**	-0.317

Table S2 - Results from means comparison tests between the age groups, OA and YA, testing for differences in parameters of target-locked neural indices. OA showed a significantly lower post-target SSVEP slope, lower pre-target alpha CoV, and lower near-response LHB slope (p < 0.05). [a]*Welch's T-Test*

(a)(iv)

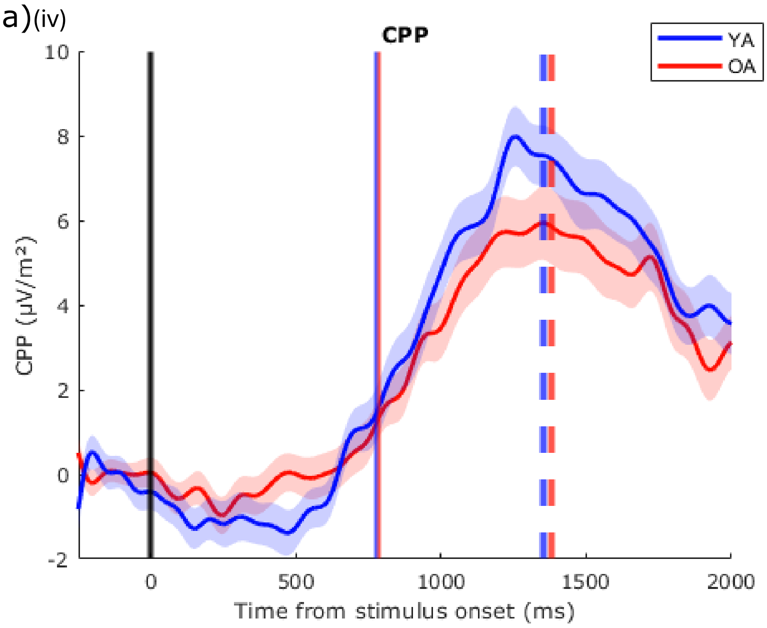

(b)(v)

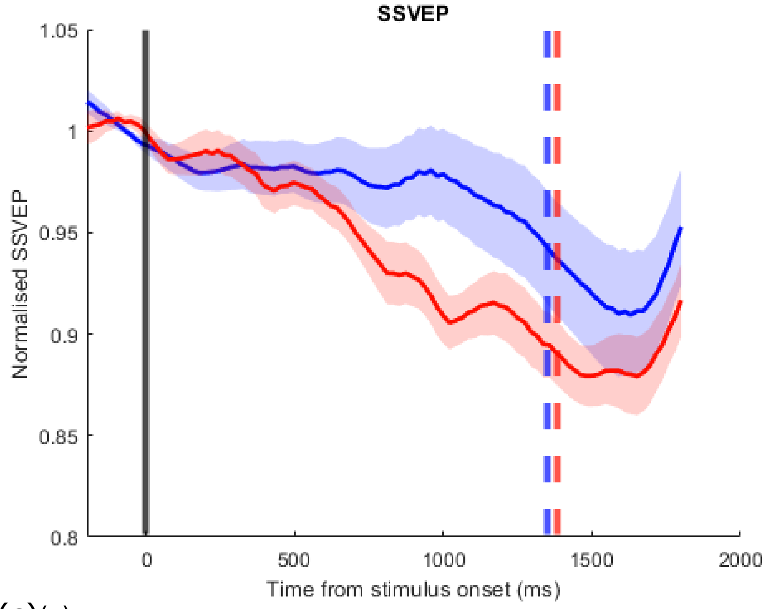

(c)(a)

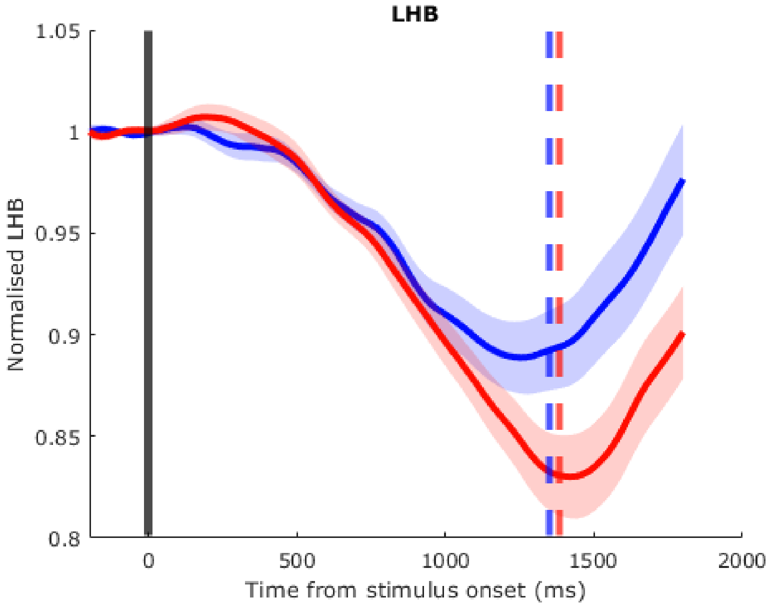

(d)(b)

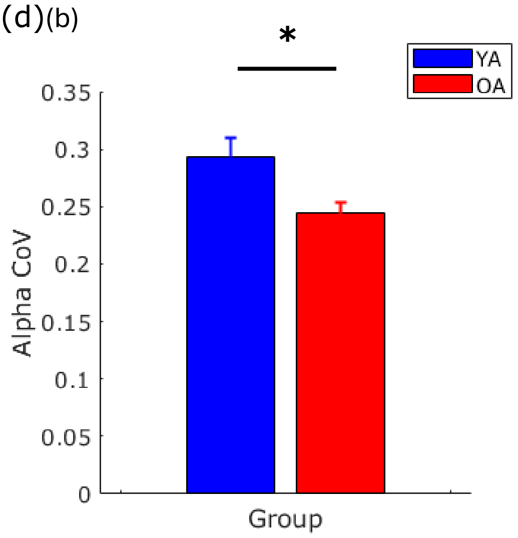

Figure S1 - Grand average target-locked neural indices (a), (b), and (c) OA shown in red, YA in blue, lighter shading shows standard error of the mean (SEM); solid black vertical line represents time of target presentation; dashed red and blue vertical lines represent respective group mean reaction time (RTm).

(a) - CPP derived from electrode A4 - indicates decision formation. Solid red and blue lines show the onset latency of the positivity which was highly similar between groups. The peak and slope were also similar.

(b) - SSVEP derived from electrode A22 from OA and A30 from YA - indicates sensory encoding of the stimulus fading in contrast. The slope (350 - 850 ms) was significantly more negative for the OA vs YA (p = 0.016).

(c) - LHB derived from electrode D19 - indicates motor preparation. The OA showed a significantly more negative slope in the 300 ms proceeding the RTm (p = 0.006).

(d) - Between-trial, pre-target alpha CoV derived from electrodes A9, A10, B7, and B8 for both groups - indicates variability in attention. Error bars indicate SEM. Alpha CoV was significantly higher in YA (p = 0.032), indicated on graph with '*'.

Pupil Diameter

With regards to post-target, target-evoked pupil diameter measures, OA had a significantly higher mean amplitude, $M = 0.06 \pm 0.02$, YA $M = -0.01 \pm 0.02$, $t(65) = 2.52$, $p = 0.01$, $d = 0.62$, and a higher peak amplitude, OA $M = 0.39 \pm 0.04$, YA $M = 0.20 \pm 0.03$, $t(65) = 3.63$, $p < 0.001$, $d = 0.90$ (Table S3). These findings are consistent with Moran's (2021, 2025, in press), however they also found that OA had a significantly lower, and more positively sloped pre-target pupil diameter than YA, which was absent in our analysis. Instead, our pre-target pupil diameter plots showed high variation (Figure S2).

Target-Locked Pupil Diameter Indices

Independent Samples T-Test

	t	**df**	***p***	**Cohen's *d***
Pre-Target pupil diameter Mean Amp	-1.164	65	0.295	-0.260
Pre-Target pupil diameter Slope	0.908	65	0.325	0.245
Post-Target pupil diameter Mean Amp	2.529	65	**0.014**	0.624
Post-Target pupil diameter Peak Amp	3.536	65	**<0.001**	0.895

Table S3 – Results from means comparison tests between the age groups, OA and YA, testing for differences in target-locked pupil diameter amplitude (amp) and slope. The OA showed a significantly greater post-target pupil diameter mean amp ($p < 0.05$) and peak amp ($p < 0.001$).

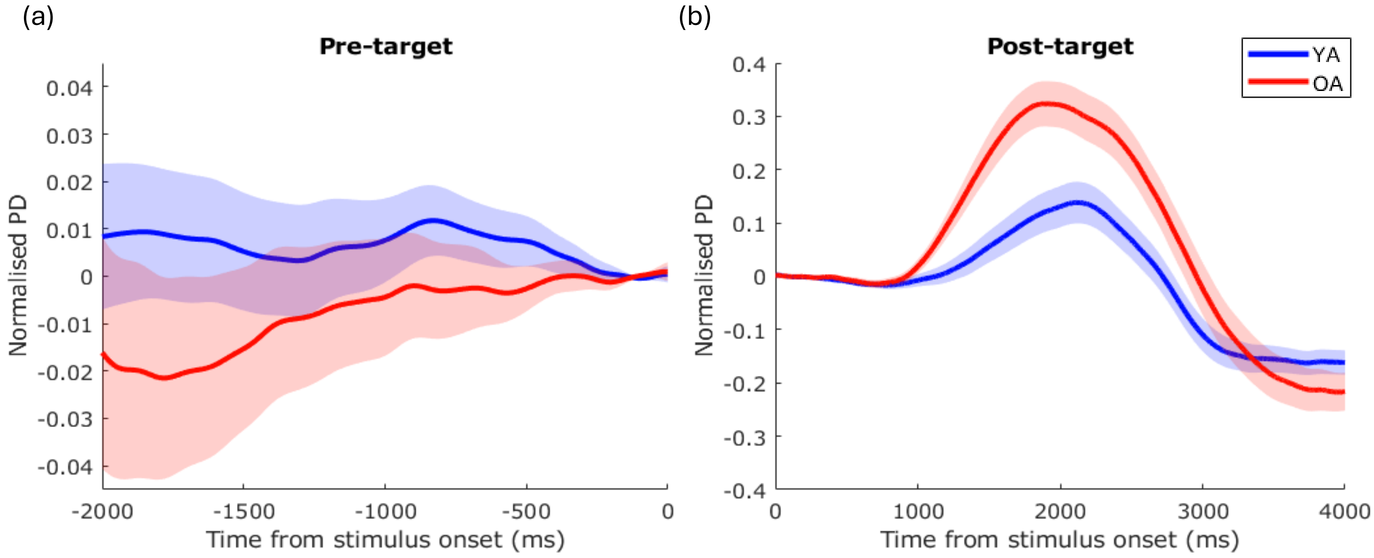

Figure S2 - Grand-averaged, normalised, target-locked pupil diameter for both groups, OA (red), and YA (blue). Shaded area indicates standard error of the mean (SEM). (a) Pre-target pupil diameter appeared to show relatively high variability, with no significant differences between the amplitude or slope. (b) Post-target pupil diameter showed an evoked response peaking at around 2000 ms. The OA had a significantly higher mean ($p = 0.014$) and peak ($p < 0.001$) amplitude.

Trial numbers contributing to physiological indices analyses

**Index**	**OA *M***	**YA *M***
CPP, SSVEP, LHB	199 ± 17	193 ± 17
Pre-target pupil diameter	306 ± 12	331 ± 7
Post-target pupil diameter	308 ± 11	329 ± 9

Table S4 – Mean number of valid trials that each participant had to contribute to the respective target-locked physiological index. Values are given as means (*M*) ± the standard error of the mean (SEM). There were 384 targets over the total task, and we deemed these means to provide contribute meaningful participant averages for each index.

Additional Supplementary Analyses

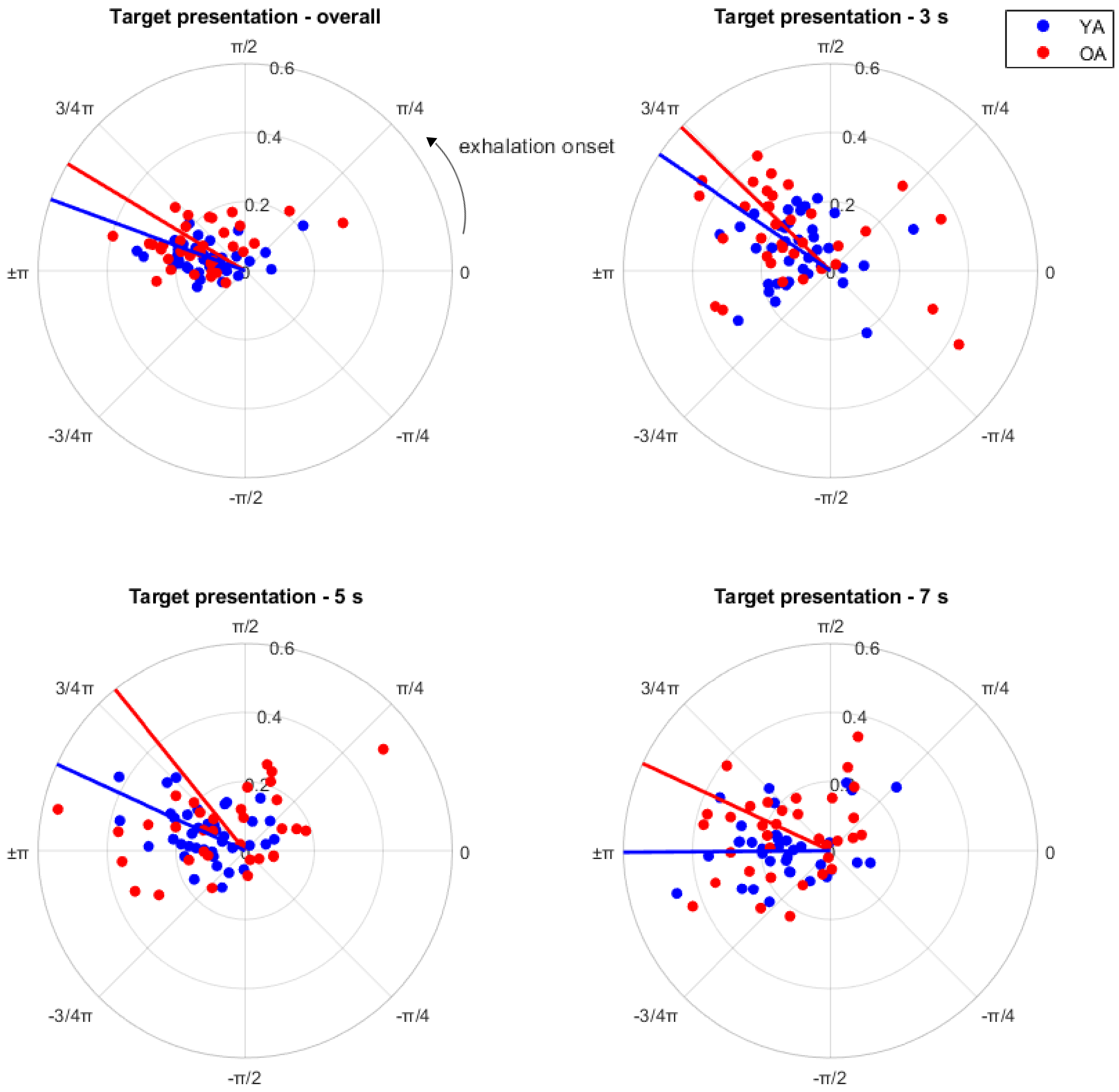

Figure S3 - Polar scatter graphs of the mean resultant vector lengths (VLs; radial axis) and respiratory phase angles (angular axis in radians), indicating the strength and angle of respiratory-task event phase-locking respectively. Top left represents respiratory phases at all targets, others are split by the inter-target intervals, 3, 5, and 7 s. Dots represent participant means and solid lines represent the direction of the group mean angles, older adults (OA) in red, younger adults (YA) in blue. 0 radians represents exhalation onset, and the respiratory cycle proceeds anti-clockwise. The majority of respiratory phase angles occurred between mid-exhalation and early inhalation. Phase-locking strength was similar for each ITI, but mean angles did significantly differ 3 vs 7 s and 5 vs 7 s.

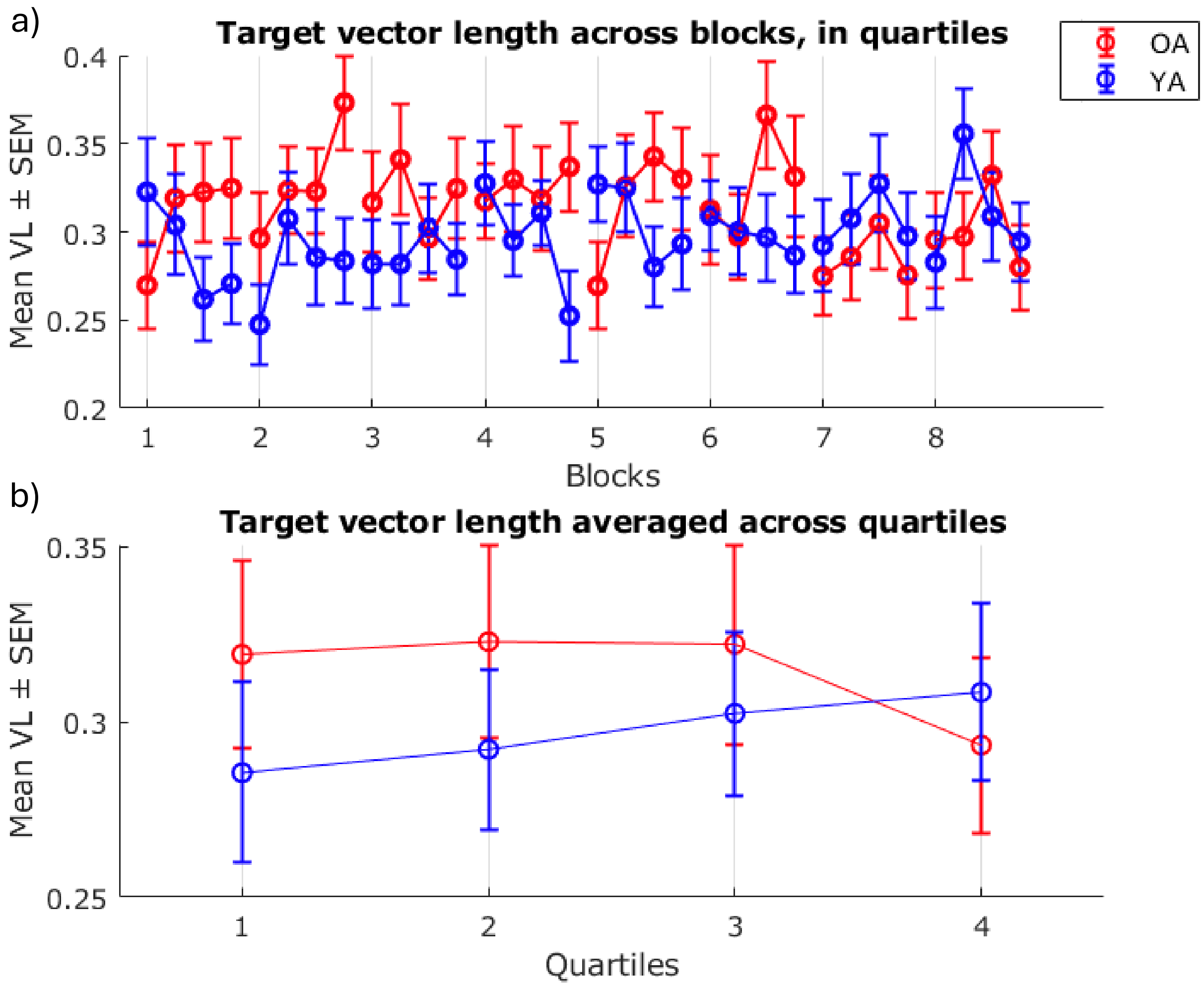

Figure S4 - Target presentation vector lengths (VL), representing the degree of respiratory-target phase-locking, (a) over the 8 blocks for each quartile, and (b) averaged across blocks for each quartile, of the GradCCD-ES task. Older Adult (OA) group shown in red and Younger Adult (YA) group shown in blue. Error bars represent the standard error of the mean (SEM). There was no discernible trend in target VL over the blocks or task.

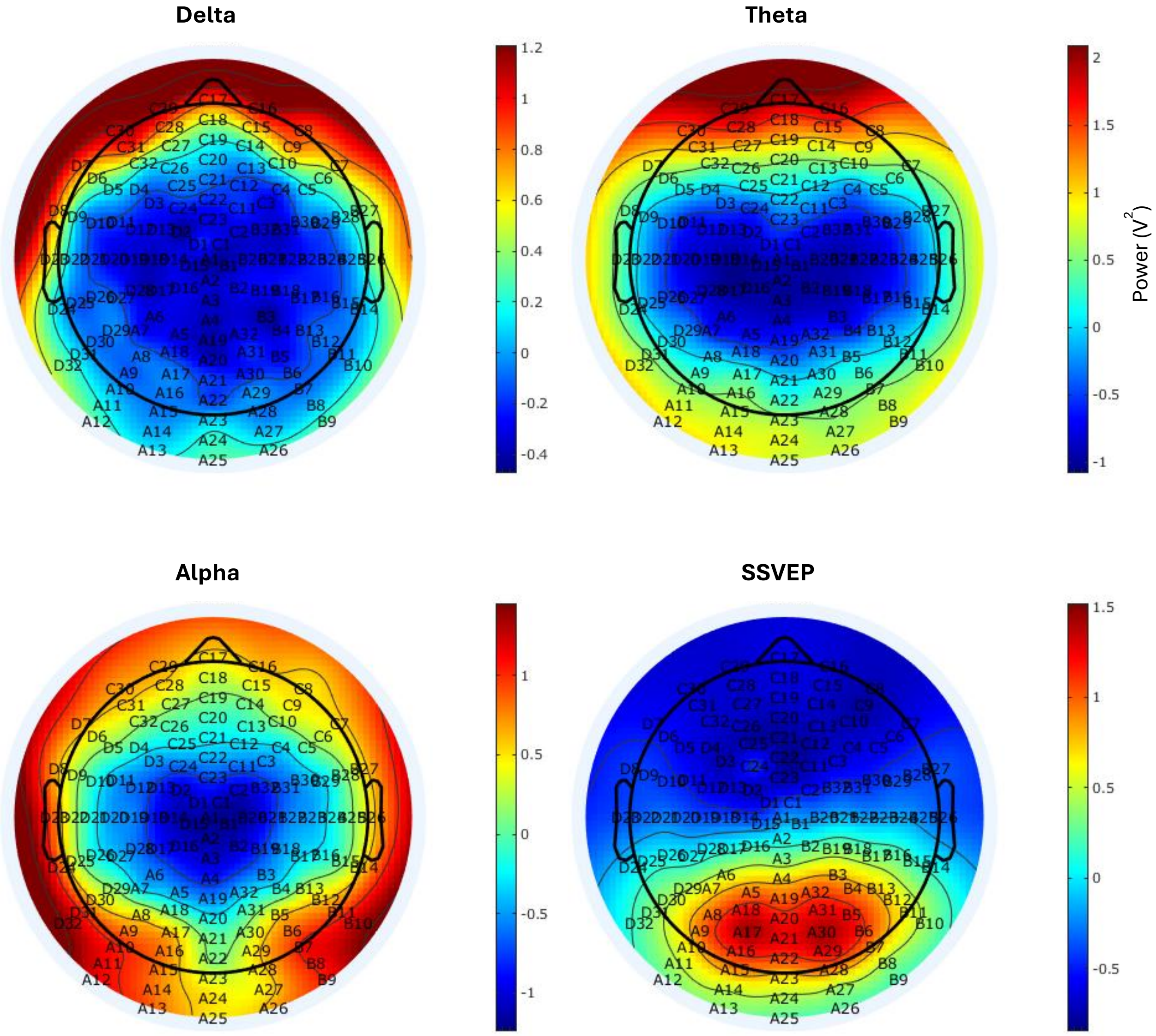

Figure S5 - Topographic plots of grand averaged power for frequency bands across the whole task. For the analysis in Figure 8, these plots informed of electrodes exhibiting maximal power. For delta (1 - 4 Hz) and theta (4 - 7 Hz), electrodes C16, C17 and C29 were selected. For alpha (8 - 14 Hz), A11, B10 and D32, and for the SSVEP (25 Hz), A17 and A30.
